# Astrocytic Slc4a4 regulates blood-brain barrier integrity in healthy and stroke brains via a CCL2-CCR2 pathway and NO dysregulation

**DOI:** 10.1016/j.celrep.2024.114193

**Published:** 2024-05-05

**Authors:** Qi Ye, Juyeon Jo, Chih-Yen Wang, Heavin Oh, Jiangshan Zhan, Tiffany J. Choy, Kyoung In Kim, Angelo D’Alessandro, Yana K. Reshetnyak, Sung Yun Jung, Zheng Chen, Sean P. Marrelli, Hyun Kyoung Lee

**Affiliations:** 1 Department of Pediatrics, Section of Neurology, Baylor College of Medicine, Houston, TX 77030, USA; 2 Jan and Dan Duncan Neurological Research Institute, Texas Children’s Hospital, Houston, TX 77030, USA; 3 Department of Biotechnology and Bioindustry Sciences, National Cheng Kung University, Tainan 70101, Taiwan; 4 Cancer and Cell Biology Program, Baylor College of Medicine, Houston, TX 77030, USA; 5 Department of Biochemistry and Molecular Genetics, University of Colorado School of Medicine, Aurora, CO 77030, USA; 6 Physics Department, University of Rhode Island, Kingston, RI 02881, USA; 7 Department of Biochemistry and Molecular Pharmacology, Baylor College of Medicine, Houston, TX 77030, USA; 8 Department of Biochemistry and Molecular Biology, The University of Texas Health Science Center at Houston, Houston, TX 77030, USA; 9 Department of Neurology, McGovern Medical School, The University of Texas Health Science Center at Houston, Houston, TX 77030, USA; 10 Department of Neuroscience, Baylor College of Medicine, Houston, TX 77030, USA; 11 Lead contact

## Abstract

Astrocytes play vital roles in blood-brain barrier (BBB) maintenance, yet how they support BBB integrity under normal or pathological conditions remains poorly defined. Recent evidence suggests that ion homeostasis is a cellular mechanism important for BBB integrity. In the current study, we investigated the function of an astrocyte-specific pH regulator, Slc4a4, in BBB maintenance and repair. We show that astrocytic Slc4a4 is required for normal astrocyte morphological complexity and BBB function. Multi-omics analyses identified increased astrocytic secretion of CCL2 coupled with dysregulated arginine-NO metabolism after Slc4a4 deletion. Using a model of ischemic stroke, we found that loss of Slc4a4 exacerbates BBB disruption, which was rescued by pharmacological or genetic inhibition of the CCL2-CCR2 pathway *in vivo*. Together, our study identifies the astrocytic Slc4a4-CCL2 and endothelial CCR2 axis as a mechanism controlling BBB integrity and repair, while providing insights for a therapeutic approach against BBB-related CNS disorders.

## In brief

Ye et al. highlight astrocytic Slc4a4’s role in astrocyte and BBB function under both normal and stroke conditions. Their study further emphasizes the significance of the Slc4a4-CCL2-CCR2 axis in BBB repair post stroke, coupled with arginine-NO metabolism.

## Graphical Abstract

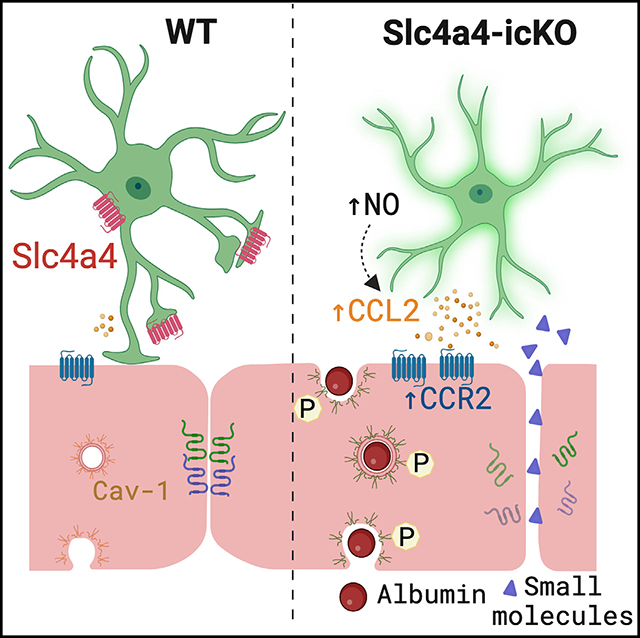

## INTRODUCTION

Disruption of blood-brain barrier (BBB) integrity is a devastating pathophysiological hallmark shared by various neurological disorders, often followed by severe consequences such as brain edema, neuronal damage, and eventually behavioral deficits.^1,2^ Therefore, understanding the mechanisms underlying BBB integrity and developing strategies to reverse BBB damage will have far-reaching impacts on neurological diseases. Among the disorders accompanied by BBB dysfunction, ischemic stroke is a leading cause of death and disability worldwide.^3^ Although the primary aim of current stroke therapy is to restore cerebral blood flow, BBB dysfunction and associated secondary brain impairments remain difficult to treat,^4^ and the mechanisms underlying stroke-induced BBB disruption are still obscure.

While the core anatomic entities comprising the BBB are tightly connected endothelial cells, emerging evidence points to a crucial role of supporting cells in the neurovascular unit in BBB function.^1,5,6^ Astrocytes are the most abundant and diverse glial cells in the central nervous system (CNS),^7,8^ and the interactions between astrocytic endfeet and blood vessel endothelial cells are essential for cerebrovascular maturation^9^ and BBB integrity.^10,11^ In particular, astrocytes secrete many paracrine factors supporting endothelial function.^12^ Among them, chemokines and cytokines, via their downstream signaling pathways, play a critical role in the recruitment and migration of leukocytes into the brain during stroke and other disease conditions.^13^ There is now mounting evidence indicating that these signaling molecules may also directly modulate BBB permeability.^14^ While the unique anatomic feature and secretome-mediated inflammatory response of astrocytes are critical for BBB function, the regulatory mechanisms underlying astrocyte-BBB interaction in healthy and stroke brains remain poorly defined.

Among the growing list of pro-inflammatory chemokines involved in stroke, CCL2 (also known as MCP1) and its receptor CCR2 are particularly noteworthy because emerging clinical evidence suggests a positive correlation between the severity of stroke progression and CCL2 levels from patients’ serum and/or cerebrospinal fluid.^15^ However, the role of CCL2 in BBB damage after stroke remains obscure, and the exact regulatory mechanism of CCL2 production is not defined. The surge of inflammatory factors after ischemic stroke is often accompanied by metabolic dysregulation.^16^ While a recent clinical study revealed a significant correlation between arginine metabolism dysregulation and CCL2 production in stroke patients,^17^ the molecular mechanism coupling the arginine metabolism pathway and CCL2 production is unknown.

To provide a favorable cellular environment, the extracellular and intracellular pH in the brain is strictly maintained by the balance of several ion species and the dynamic regulation of multiple ion transporters.^18^ Conversely, dysregulation of pH homeostasis in the brain is often implicated in a range of neurological disorders.^19–21^ CO_2_/HCO_3_^−^ is one of the most abundant and effective buffering ion pairs for pH control,^18^ and the imbalance of CO_2_/HCO_3_^−^ contributes to drastic pH reduction after ischemic stroke injury.^22^ Previous literature has demonstrated that astrocyte-enriched electrogenic sodium-bicarbonate cotransporter 1 (Slc4a4) is responsible for shuttling HCO_3_^−^ across the cell membrane in a bidirectional manner to regulate both intra- and extracellular pH in response to intracellular signaling cascades as well as extracellular pH.^23–26^ In the clinical setting, Slc4a4 variants in patients are associated with a number of CNS disorders, including ischemic stroke.^27–31^ Despite its central role as an astrocytic pH regulator, whether and how Slc4a4 governs astrocyte-endothelial cell interaction in BBB maintenance and repair after stroke has not been investigated.

Here, we employed conditional genetic mouse models to investigate the role of Slc4a4 in both health and disease. Temporal ablation of Slc4a4 in astrocytes illustrated a requisite role of Slc4a4 in astrogenesis, astrocyte morphological complexity, and astrocytic Ca^2+^ physiology during normal development. In addition, loss of astrocytic Slc4a4 resulted in abnormal BBB structure and function in both normal and ischemic stroke brains. To identify mechanisms by which astrocytic Slc4a4 regulates BBB function, we performed unbiased multi-omics analyses of Slc4a4-deficient mice cortices and conditional media of Slc4a4-ablated astrocytes and discovered increased astrocytic CCL2 secretion upon loss of astrocytic Slc4a4. Moreover, our metabolomics profiling of Slc4a4-deficient cortices indicated that dysregulated arginine-nitric oxide (NO) metabolism is associated with increased CCL2 production. Further studies using either pharmacological or genetic inhibition of CCL2 and NO demonstrated the role of the Slc4a4 in regulating the CCL2-CCR2 pathway and NO metabolism in the context of BBB recovery after stroke. Together, our study elucidates an indispensable role of Slc4a4 in astrocyte-BBB interaction, highlighting glial ion regulators as therapeutic targets for BBB-related pathology in ischemic stroke.

## RESULTS

### Loss of astrocytic Slc4a4 reduces astrocyte morphological complexity in the brain

**Figure 1.**
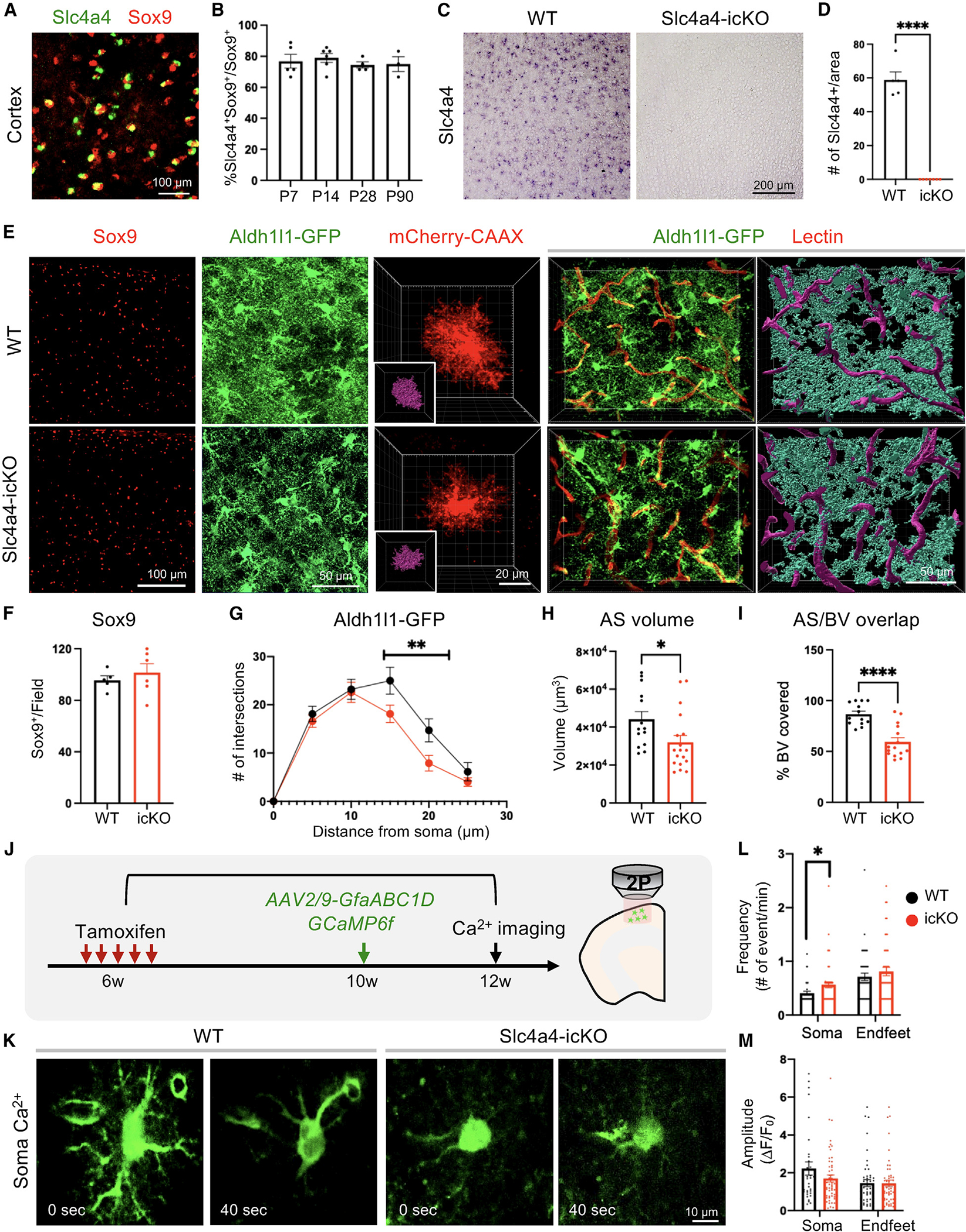
Slc4a4 is required for morphological complexity and proper Ca^2+^ propagation in the adult brain (A) Double *in situ*-immunofluorescence staining of Slc4a4 in astrocyte lineage (Sox9) in P28 mouse cortex. (B) Quantification of the number of Slc4a4-expressing cortical astrocytes (Slc4a4+Sox9+). Each data point represents an individual animal. *n* = 3–5 animals for each age. (C and D) *In situ* hybridization confirms the deletion of Slc4a4 in the cortex. Each data point represents an individual animal. *n* = 6 per genotype. (E) Representative immunofluorescence images of astrocyte markers (Sox9, Aldh1l1-GFP) in the cortex at P90. Astrocyte morphology is labeled at single-cell resolution using AAV-PhP.eB-GfaABC_1_D-mCherry-CAAX. Blood vessels were labeled by td-tomato lectin (red). Astrocyte-blood vessel interactions were reconstructed using IMARIS. (F) Quantification of the number of Sox9+ cells in the cortex at P90. Each dot indicates an individual animal. (G) Overall complexity of astrocytes (Aldh1l1-GFP) was measured by Sholl analysis. *n* = 24–36 cells collected from 4–6 mice per genotype. **p < 0.01 by two-way ANOVA. (H) Astrocyte volume was reconstructed and quantified using IMARIS software. Each dot represents an individual astrocyte. *n* = 14–18 astrocytes collected from 4 mice per group. (I) Quantification of blood vessel area covered by astrocytes after IMARIS 3D reconstruction. Each dot represents an individual section. *n* = 13–17 sections collected from 4–5 mice per genotype. ****p < 0.0001 by Student’s t test. (J) Schematic of measuring astrocytic spontaneous Ca^2+^ signaling. (K) Representative images of astrocytic soma spontaneous Ca^2+^ activity. (L and M) Quantification of frequency (L) and amplitude (M) of GCaMP6f signal events in astrocyte soma and endfeet. Each dot represents an individual cell. *n* = 30–40 cells collected from 6–8 mice of each genotype. *p < 0.05 by Student’s t test. All data are presented as mean ± SEM.

As a first step toward investigating the role of Slc4a4 in astrocytes, we assessed its expression in the mouse brain. We found that Slc4a4 mRNA was expressed in the majority of astrocytes (Sox9+) in the cortex, but not neurons (NeuN+) (Figures 1A and S1A). Our analysis further revealed that >70% of Sox9-expressing astrocytes co-expressed Slc4a4 in the cortex during development and throughout adulthood (Figures 1B and S1B).

To delineate how Slc4a4 functions in astrocyte development, we generated astrocyte-specific Slc4a4 conditional null mice by crossing Slc4a4-floxed (Slc4a4^F/F^) mice with an Aldh1l1-CreER driver line^32^ (Aldh1l1-CreER; Slc4a4^F/F^, denoted as Slc4a4-icKO). We administered tamoxifen either during development (injection at P3 and harvested at P30) or in adulthood (injection at 6 weeks and harvested at 12 weeks) for analysis, confirming complete deletion of Slc4a4 in the cortex by *in situ* staining (Figures 1C and 1D) and by real-time qPCR on fluorescence-activated cell (FAC)-sorted astrocytes (Figure S1C). Compared to wild-type (WT) littermates, mice with Slc4a4 deletion during development, but not in adulthood, showed a 35% reduction in the number of Sox9+ astrocytes (Figures S1D, S1F, 1E, and 1F).

To visualize the morphological complexity of astrocytes, we further crossed Slc4a4-icKO mice with the Aldh1l1-EGFP reporter line^33^ and performed high-resolution confocal imaging for GFP+ astrocytes. Sholl analysis (IMARIS software) revealed that Aldh1l1-GFP+ astrocytes from Slc4a4-icKO mice had markedly decreased branches compared with controls throughout brain development (up to 50% reduction) and adulthood (up to 28% reduction) (Figures S1D, S1G, 1E, and 1G). To assess the morphological changes of Slc4a4-depleted astrocytes more precisely, we sparsely labeled cortical astrocytes with an adeno-associated virus (AAV) vector expressing membrane-targeted mCherry under the astrocyte-specific promoter GfaABC_1_D (AAV-PhP.eB-GfaABC_1_D-mCherry-CAAX),^34^ followed by tamoxifen-induced Slc4a4 deletion during early development or adulthood. In both cases, we found that cortical astrocytes had a significantly smaller volume (28% reduction) after Slc4a4 deletion compared to WT (Figures S1D, S1H, 1E, and 1H). Since the unique, complex, and bushy morphological features of astrocytes are required for their extensive contact with endothelial cells,^35^ we measured the interaction between astrocytes and blood vessels using an Aldh1l1-GFP reporter and fluorescent lectin. We found that the reduction in branched morphology of cortical astrocytes in adult Slc4a4-icKO mice was coupled with a 32% decrease in the vasculature area covered by astrocytic endfeet processes, validated by 3D rendering (Figures 1E and 1I). Together, these data indicate that Slc4a4 is required for astrocyte morphology and its association with blood vessels in the cortex.

### Slc4a4-depleted astrocytes exhibit abnormal Ca^2+^ wave propagation

Previous studies have suggested a correlation between astrocytic morphological complexity and calcium (Ca^2+^) dynamics in the soma and processes, a direct indication of astrocyte physiological activity.^36^ To address whether astrocytic Ca^2+^ activity is altered in the absence of Slc4a4, we used an AAV vector to express a genetically encoded Ca^2+^ indicator (AAV2/9-GfaABC1-GCaMP6f)^37^ in the cortex of adult Slc4a4-icKO and WT mice (Figure 1J). Two weeks after viral injection, we used established imaging algorithms to assess Ca^2+^ dynamics in the soma and endfeet of cortical astrocytes. While Ca^2+^ amplitudes in Slc4a4-deficient astrocytes remained unchanged in both soma and endfeet compared to WT astrocytes, we observed a slight increase (20%) of Ca^2+^ frequency in the soma, but not endfeet, of Slc4a4-deficient astrocytes (Figures 1J–1M, and S1E). Together, these data indicate that loss of Slc4a4 moderately alters astrocyte Ca^2+^ physiology in the cortex.

### Loss of Slc4a4 results in hyper-vascularization coupled with junctional marker loss at the BBB

**Figure 2.**
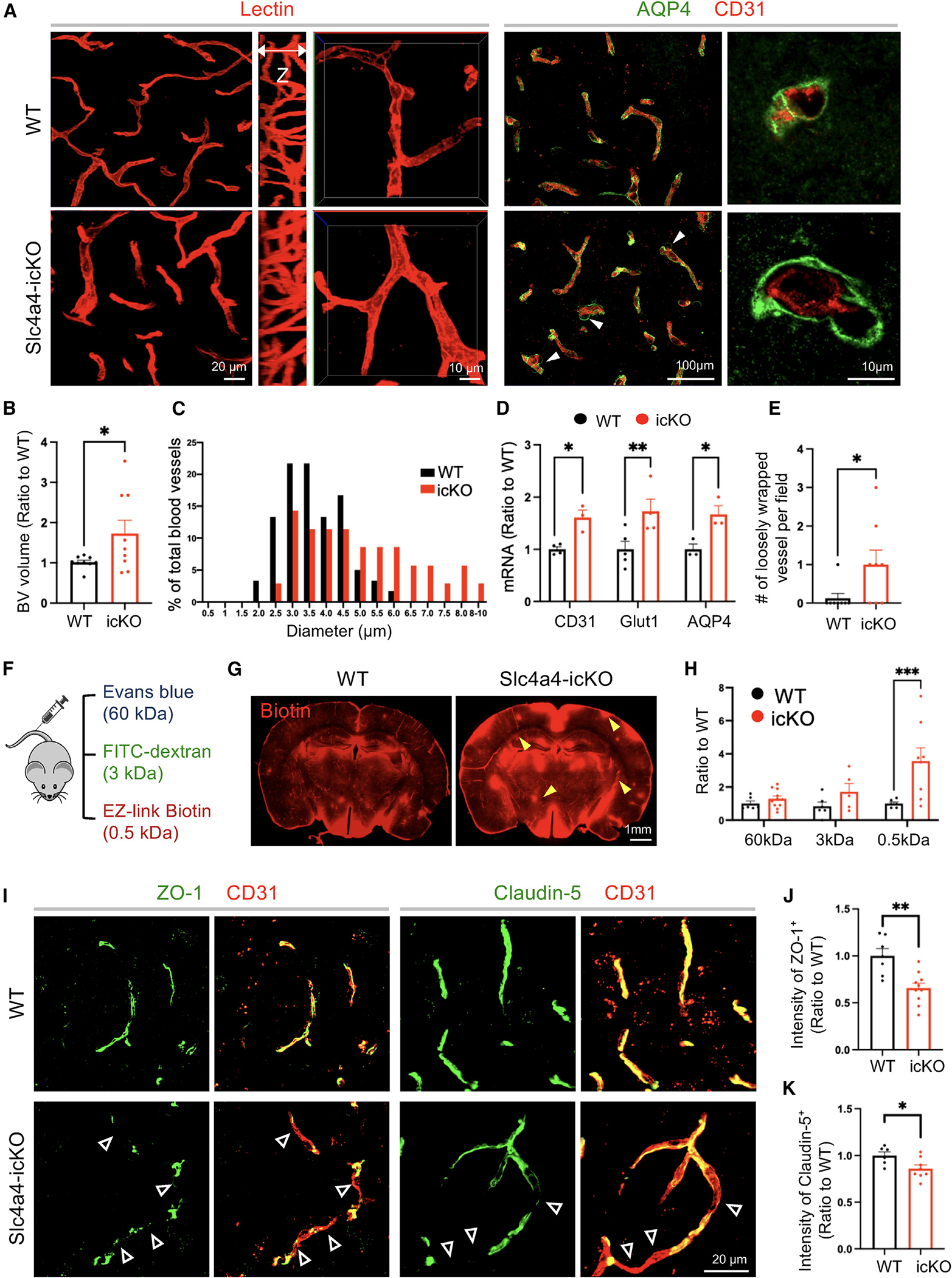
Loss of Slc4a4 results in hyper-vascularization coupled with BBB leakage (A) Blood vessel phenotype in the cortex at P90 was examined by *in vivo* lectin labeling and immunofluorescence staining of vasculature marker CD31. Astrocytic endfeet were labeled by AQP4. (B) Quantification of blood vessel volume from IMARIS 3D reconstruction. Each dot represents an individual section. *n* = 9 sections and collected from *n* = 3–4 animals per genotype. *p < 0.05 by Student’s t test. (C) A histogram of average blood diameter measurements in each genotype. *n* = 35–60 vessels collected from 4–5 animals per genotype. (D) qRT-PCR analysis of endothelial cell markers in the cortex. Each dot represents an individual animal. *n* = 3–5 animals per genotype. *p < 0.05, **p < 0.01 by Student’s t test. (E) Quantification of the number of loosely wrapped endfeet structures. Each dot represents an individual animal. *n* = 3–5 animals per genotype. *p < 0.05 by Student’s t test. (F) BBB leakage was assessed by Evans blue (indicates albumin leakage), fluorescein isothiocyanate (FITC)-conjugated dextran (3 kDa), and EZ-Link Sulfo-NHS-Biotin (indicates small-molecule leakage). (G) Representative images of stained biotin in the cortex. Yellow arrowheads indicate leakage of EZ-Link Sulfo-NHS-Biotin into the brain. (H) Extravasated Evans blue levels were quantified by colorimetric assays. Extravasated FITC-dextran and EZ-Link Sulfo-NHS-Biotin were quantified based on intensity in brain sections. Each dot represents an individual animal. *n* = 6–8 per genotype. ***p < 0.001 by Student’s t test. (I) Double immunofluorescence staining of tight-junction markers (ZO-1, Claudin-5) and endothelial cell marker (CD31) in the cortex. Empty arrowheads indicate vessels missing coverage by tight-junction proteins. (J and K) Quantification of the intensity of ZO-1 and Claudin-5 colocalized with CD31. Each dot represents an individual blood vessel. *n* = 6–8 vessels collected from 3 animals per genotype. *p < 0.05, **p < 0.01 by Student’s t test. All data are presented as mean ± SEM.

To investigate whether the observed morphological and physiological changes in Slc4a4-ablated astrocytes influence BBB function, we next evaluated whether the brain vasculature was altered in the absence of astrocytic Slc4a4. Using lectin to label blood vessels *in vivo*, we observed a 43% increase in blood vessel volume in Slc4a4-icKO mice compared to WT control (Figures 2A and 2B) and a shift of blood vessel diameter distribution toward larger-sized vessels in Slc4a4-icKO mice (Figure 2C). Consistent with this enlarged blood vessel phenotype, we saw an up to 2-fold increase in endothelial cell marker expression (CD31 and Glut1) (Figures 2D and S2A–S2C). Notably, we observed a 50% upregulation of AQP4 expression in Slc4a4-icKO mice (Figures 2A, 2D, S2A, and S2D), a water channel known to be upregulated in response to osmotic imbalance by abnormal intracellular ion concentrations (e.g., Na^+^).^38^ Despite the upregulation of AQP4 expression, we observed an increased number of loosely wrapped astrocyte endfeet around blood vessels (Figures 2A and 2E). To investigate if enlarged cortical blood vessels in Slc4a4-icKO affect BBB function, we performed BBB leakage experiments *in vivo* by injecting molecules of different sizes (Figure 2F). Although no significant leakages of albumin (Evans blue) and 3-kDa dextran were observed, we found a more than 3-fold increase of small molecules infiltrated into the brain parenchymal (MW = 0.5 kDa, EZ-Link Sulfo-NHS-Biotin) (Figures 2G, 2H; S2E). Since biotins are known to diffuse into the brain parenchyma via the paracellular pathway upon endothelial tight-junction disruption,^39^ we examined whether loss of astrocytic Slc4a4 affected the expression of endothelial tight-junction markers and found a deficient coverage of blood vessels (CD31^+^) by either ZO-1 or Claudin-5 (35% and 15% decrease, respectively) (Figures 2I–2K) in Slc4a4-icKO cortices. We also observed an 18% reduction of VE-Cadherin expression in Slc4a4-icKO cortex (Figures S2F and S2G). Collectively, these data suggest a critical role of astrocytic Slc4a4 in regulating endothelial tight and adherens junctions and BBB paracellular transport in the adult brain.

### Slc4a4-deficient astrocytes exhibit impaired BBB remodeling after ischemic stroke

**Figure 3.**
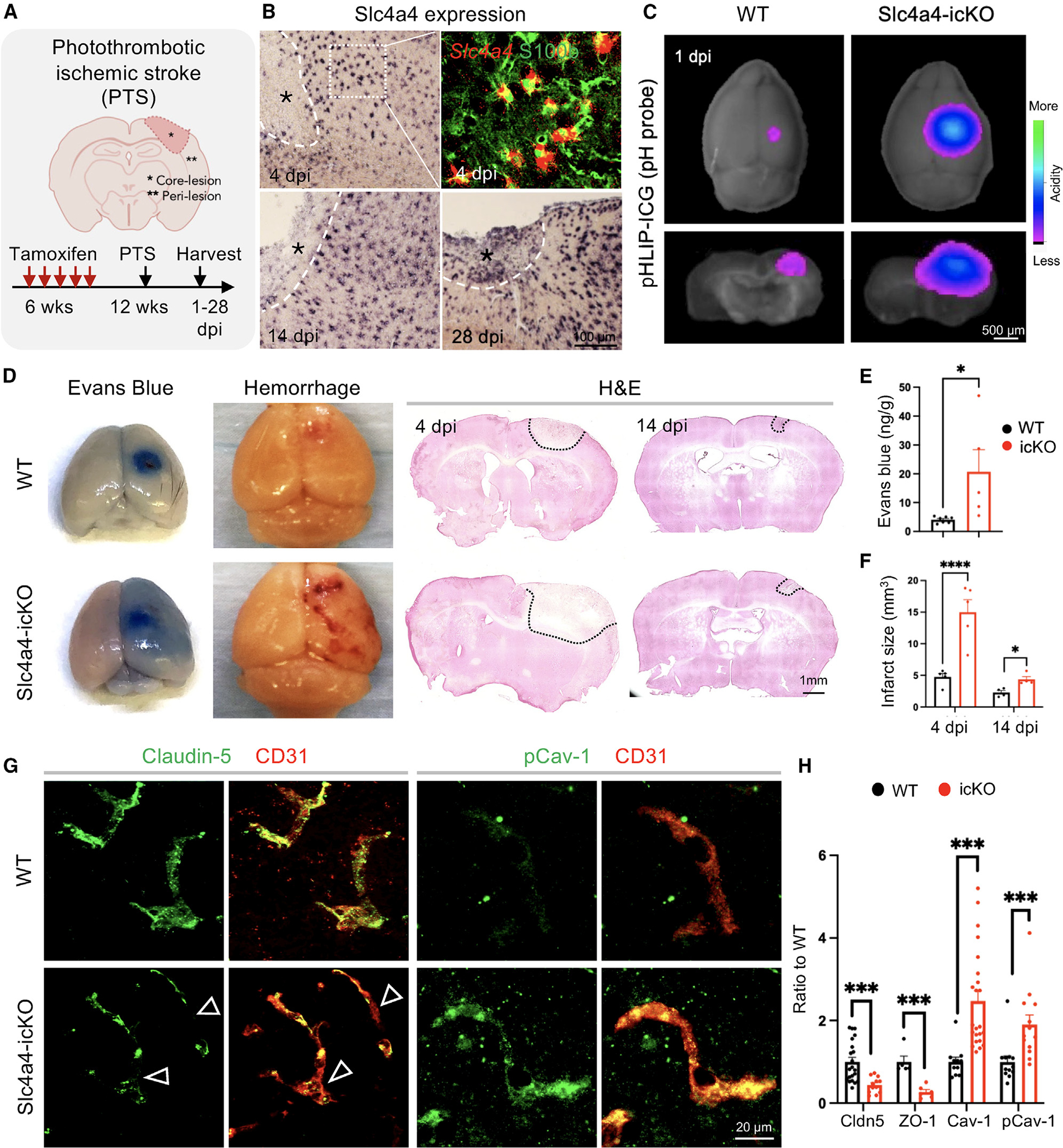
Slc4a4-deficient astrocytes exhibit impaired BBB remodeling after ischemic stroke (A) Schematics of photothrombotic ischemic stroke (PTS) in WT and Slc4a4-icKO mice. Peri-lesion is defined as a 150-mm distance from the lesion border. (B) Single or double staining of *Slc4a4* (*in situ*) and S100b (immunostaining) in the WT cortex after PTS. (C) Extracellular pH in the stroke lesions was measured by pHLIP-ICG dye (1 mg/kg). (D) Representative images of albumin leakage (Evans blue), hemorrhage, and gross histology (H&E) at 4 or 14 dpi. (E) Evans blue levels were determined by colorimetric assays. Each dot represents an individual animal. *n* = 5–7 per genotype. *p < 0.05 by Student’s t test. (F) Quantification of infarct size is based on H&E staining of serial 40-mm-thick brain sections at 4 and 14 dpi. Each dot represents an individual animal. *n* = 4–6 per genotype per time point. *p < 0.05, ****p < 0.0001 by Student’s t test. (G) Double immunofluorescence staining of tight-junction marker Claudin-5 and caveolae marker pCav-1 with endothelial cell marker (CD31) at the peri-lesion area at 4 dpi. Empty arrowheads indicate loss of Claudin-5 (Cldn5). (H) Quantification of tight-junctional markers (Cldn5, ZO-1) and caveolae markers (Cav-1, pCav-1) based on their intensity colocalized with CD31 in immunostaining. Each dot represents an individual blood vessel. *n* = 7–20 blood vessels collected from 3–6 animals per genotype with at least two vessels per animal. ***p < 0.001 by Student’s t test. All data are presented as mean ± SEM.

To determine whether astrocytic Slc4a4 contributes to BBB remodeling after injury, we employed a cortical photothrombotic stroke (PTS) model (Figure 3A). We first found that Slc4a4 was highly expressed in S100b+ reactive astrocytes, suggesting its potential role after ischemic stroke (Figure 3B). We confirmed that Slc4a4 remains deleted in all the astrocytes in Slc4a4-icKO around the lesion area after stroke at 4 days post-injury (dpi) (Figure S3A). Next, we examined whether loss of Slc4a4 in-fluences pH homeostasis after PTS using an acidic extracellular pH-sensing probe (pH-low insertion peptide [pHLIP]) labeled with fluorescent dye ICG (pHLIP-ICG).^40^ The radius of acidic milieu measured by pHLIP-ICG was greater at 1 dpi compared with 4 dpi after PTS (Figure S3B). Furthermore, Slc4a4-icKO mice exhibited a 3-fold increase in extracellular acidity range compared to WT at 1 dpi (Figures 3C and S3C), suggesting that astrocytic Slc4a4 is required for maintaining pH homeostasis after stroke.

We next examined whether exacerbated extracellular acidity in Slc4a4-icKO mice is associated with more severe pathological presentations after stroke. Strikingly, we observed a 5-fold increase in albumin leakage into Slc4a4-icKO brains (Evans blue) along with an extensive hemorrhage area (Figures 3D and 3E) and a 3-fold increase in infarct volume at 4 dpi (H&E) (Figures 3D, 3F; S3D). The increased infarct volume in Slc4a4-icKO mice persisted until the stroke recovery stage at 14 dpi (Figures 3D and 3F). The drastic BBB leakage at 4 dpi prompted us to examine the BBB structural integrity after stroke. In the Slc4a4-icKO brains, expression of tight-junction proteins (Claudin-5, ZO-1) was reduced by 56% and 75% at the peri-lesion area compared to WT controls at 4 dpi (Figures 3G, 3H; S3E). Since blood-borne proteins, such as albumin, cross the BBB via endothelial caveolae-mediated transcytosis, we further examined the expression of caveolin-1 (Cav-1), a structure protein of caveolae, and its phosphorylated form (pCav-1), an indicator of endothelial transcytosis activity.^41^ Corresponding to increased albumin leakage in the Slc4a4-icKO brains after stroke (Figures 3D and 3E), we found a 2-fold increase in both endothelial Cav-1 and pCav-1 (Figures 3G, 3H; S3E), suggesting elevated caveolae-mediated transcytosis. In contrast. there was no difference in VE-cadherin expression between Slc4a4-icKO and WT mice (Figures S3F and S3G). Taken together, our results demonstrate that astrocytic Slc4a4 is required for re-establishing pH homeostasis and recovering BBB function after stroke.

### Loss of astrocytic Slc4a4 dampens reactive astrogliosis and astrocyte-endothelial interaction after stroke

**Figure 4.**
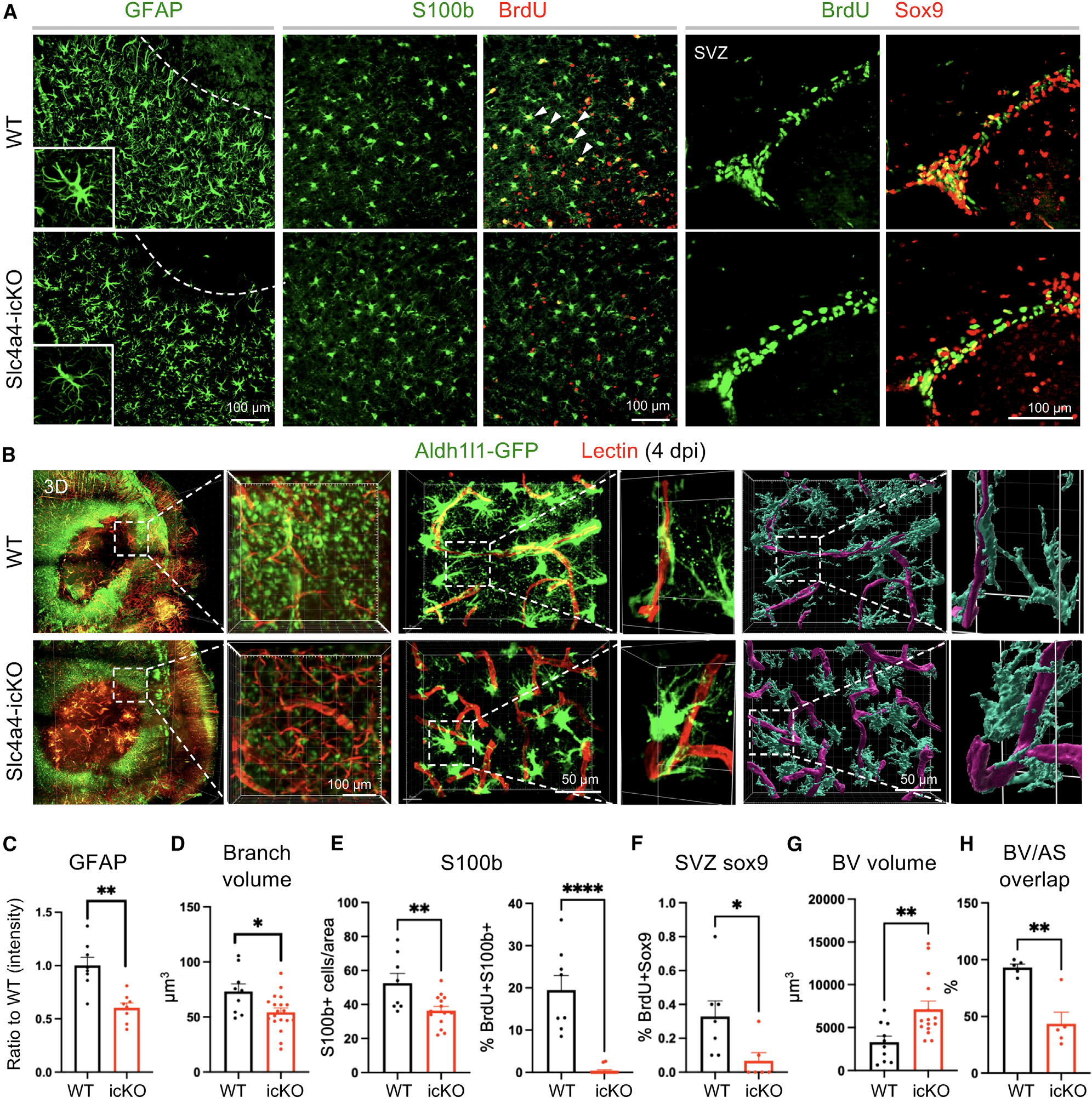
Loss of astrocytic Slc4a4 dampens reactive astrogliosis and astrocyte-BBB interaction after stroke (A) Immunostaining of reactive astrocyte markers (GFAP, S100b) at the peri-lesion area at 4 dpi. S100b+ cells are co-labeled with BrdU to indicate local astrocyte proliferation. SVZ Sox9+ cells are co-labeled with BrdU to indicate SVZ astrocyte proliferation. (B) Whole-mount images of CLARITY-cleared mice brain at 4 dpi of PTS. Astrocytes were genetically labeled with Aldh1l1-GFP (green), and blood vessels were labeled with tomato lectin (red); 40-mm-thick sections were used for further IMARIS 3D reconstruction to visualize astrocyte-blood vessel interaction. (C) Quantification of GFAP intensity from immunostaining. Each dot represents an individual animal. *n* = 8 per genotype, **p < 0.01 by Student’s t test. (D) Quantification of total branch volume of reactive astrocytes from GFAP immunostaining. *n* = 9–19 cells collected from 3–5 mice per genotype. *p < 0.05 by Student’s t test. (E) Quantification of S100b+ and S100b+ BrdU+ cell number from immunostaining. Each dot represents an individual animal. *n* = 8–14 per genotype. **p < 0.01, ****p < 0.0001 by Student’s t test. (F) Quantification of the number of proliferating SVZ astrocytes (BrdU+; Sox9+). Each dot represents an individual animal. *n* = 6–7 animals per genotype. *p < 0.05 by Student’s t test. (G and H) Quantification of blood vessel volume (G) and volume covered by astrocyte processes (H) in the peri-lesion area at 4 dpi. Each dot represents an individual animal. *n* = 5–14 per genotype. **p < 0.01 by Student’s t test. All data are presented as mean ± SEM.

Given the critical role of Slc4a4 in astrocytic interaction with endothelial cells and the key function of reactive astrocytes in BBB reconstruction after injury, we evaluated the role of Slc4a4 in reactive gliosis after stroke. Analysis of reactive astrocyte markers at 4 dpi showed a 40%–50% reduction of GFAP expression and a 25% reduction in total branch volumes in reactive astrocytes surrounding the infarct lesion in Slc4a4-icKO mice compared with WT mice at 4 dpi (Figures 4A, 4C, and 4D). In addition, we observed a 30% decrease in the overall number of reactive astrocytes (S100b+) surrounding the lesion area of Slc4a4-icKO mice at 4 dpi (Figures 4A and 4E). To label proliferating astrocytes, we administered multiple doses of bromodeoxyuridine (BrdU) (200 mg/kg, every 12 h from 1 to 3 dpi). Strikingly, we found an almost 100% reduction in proliferating astrocytes (S100b+; BrdU+) at peri-lesion areas of Slc4a4-icKO mice, indicating impaired local astrocyte proliferation after stroke. Since subventricular zone (SVZ) neural stem cells give rise to a subpopulation of reactive astrocytes in the stroke-lesioned area to participate in glial scar formation,^42^ we further investigated whether reduced SVZ astrocyte proliferation decreases the number of reactive astrocytes surrounding stroke lesions, and we found an 80% reduction in proliferating SVZ astrocytes (BrdU+; Sox9+, Figures 4A and 4F). At the chronic stage of stroke (14 dpi), we did not observe any proliferating astrocytes at the peri-lesion area or the SVZ zone in either WT or Slc4a4-icKO mice (Figure S3H). Nevertheless, we found that persistent dampened reactive gliosis, including a thinner glial scar, decreased S100b expression and reduced the number of astrocytes surrounding the lesion (Figures S3H–S3J). Together, these data indicate that loss of Slc4a4 impairs both local and SVZ astrocyte proliferation after cortical ischemia, which further leads to dampened reactive astrocyte response surrounding the lesion in both acute and chronic stages of ischemic stroke.

To test whether loss of astrocytic Slc4a4 results in defective interaction between reactive astrocytes and blood vessels after stroke, fluorescent lectin-perfused Slc4a4-icKO and WT mice brains were subjected to CLARITY-based tissue clearing followed by light-sheet microscopy. Consistent with 2D histological staining in Figures 3D, 3D reconstruction of whole-mounted brains revealed larger infarct areas in Slc4a4-icKO mice with enlarged surrounding blood vessels (Figures 4B and 4G). Of note, the blood vessel enlargement by loss of astrocytic Slc4a4 was much more pronounced after stroke compared to normal conditions (5-fold vs. 1.7-fold). Further confocal imaging of brain sections, followed by IMARIS 3D-rendering, showed a marked reduction of blood vessel area covered by astrocyte processes (53% decrease after stroke vs. 32% without stroke) (Figures 4B and 4H). This observation suggests that astrocytic Slc4a4 is critical for astrocyte-blood vessel interaction after ischemic stroke.

A previous report shows that Slc4a4 is deleted by Aldh1l1-CreER in the kidney,^43^ and we did observe a partial deletion of kidney Slc4a4 in our Slc4a4-icKO (Figure S4A). To further investigate the role of Slc4a4 in a brain-specific manner, we utilized the AAV-GfaABC1D-cre virus to achieve high specificity and efficiency in astrocytic Slc4a4 deletion without affecting kidney Slc4a4 expression (Figures S4B–S4D). Using this viral approach, we replicated key stroke-related phenotypes observed in the Slc4a4-icKO mouse line after the stroke, including enlarged infarct size (Figures S4D and S4E), impaired reactive astrocytes response (Figures S4D, S4F, and S4G), increased transcellular and paracellular transport at the BBB (Figures S4D, S4H, and S4I), and increased capillary size (Figures S4D and S4J). Collectively, our data demonstrate the necessity of astrocytic Slc4a4 in maintaining BBB functions after stroke, which is independent of kidney Slc4a4 expression.

### Slc4a4 regulates astrocyte-BBB integrity via astrocyte-derived cytokine CCL2 and endothelial CCR2

To interrogate the mechanisms by which astrocytic Slc4a4 regulates astrocyte-BBB interactions, specifically astrocyte-endothelial interactions, we focused on astrocytic secretory factors as astrocyte-derived factors are critical in angiogenesis and BBB function.^44^ We first exposed a mouse endothelial cell line (bEnd3) to conditioned medium (CM) from either WT (WT-CM) or Slc4a4-deficient astrocytes (KO-CM) (Figure S5A). We observed that endothelial cell size (CD31) was enlarged by ~60%, and the number of proliferating endothelial cells (BrdU) was increased by 2-fold in the KO-CM-treated group (Figures S5B–S5E). In addition, we found a reduction of junctional marker ZO-1 expression upon treatment with KO-CM (Figures S5B and S5E).

To directly examine whether astrocytes lacking Slc4a4 affect BBB permeability via astrocytic secretory factors, we performed co-culture assays and evaluated the transendothelial electrical resistance (TEER) across this bilayer, an established metric of paracellular BBB permeability *in vitro* (Figure S5F). Interestingly, endothelial cells co-cultured with Slc4a4-deficient astrocytes displayed increased permeability, as indicated by the decreased electric resistance (Figure S5G). This observation demonstrates that Slc4a4 regulates astrocyte-mediated maintenance of BBB integrity via paracrine signaling factors, independent of direct physical interactions with endothelial cells.

**Figure 5.**
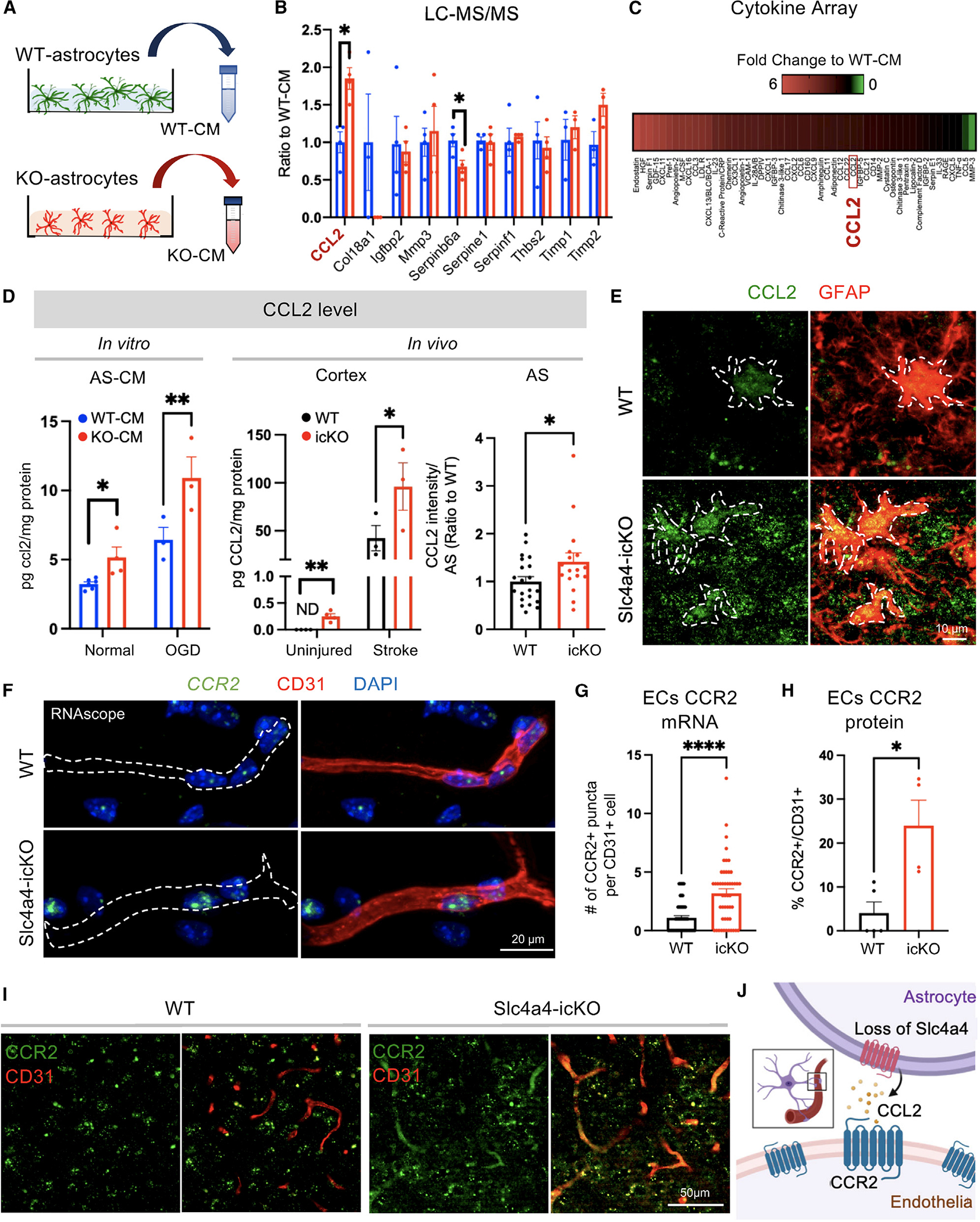
Loss of Slc4a4 upregulates expression of astrocytic CCL2 and endothelial CCR2 after ischemic stroke (A) Conditioned medium (CM) was collected from primary WT and Slc4a4 KO astrocytes and subjected to LC-MS/MS-based unbiased proteomics and cytokine/chemokine array. (B) Angiogenic factors detected from LC-MS/MS-based unbiased proteomics. (C) Cytokine/chemokines changed in the CM from WT and Slc4a4 KO astrocytes. Pooled CM from three independent cultures per genotype were used (D) CCL2 level was measured by ELISA in CM collected under either normal or oxygen-glucose-deprivation (OGD) condition cortices from uninjured and stroked (1 dpi) brains from WT and Slc4a4-icKO mice. Each dot represents CM or cortices collected from an individual animal. *n* = 3–5 per genotype. *p < 0.05, **p < 0.01 by Student’s t test. *In vivo* astrocytic CCL2 expression after stroke at 4 dpi was quantified from double immunostaining. *n* = 17–21 cells collected from 3–5 mice per genotype. *p < 0.05 by Student’s t test. (E) Representative images of astrocytic CCL2 expression at 4 dpi by double immunostaining of CCL2/GFAP. (F and G) Endothelial CCR2 mRNA expression was visualized by RNA scope and CD31 staining and quantified by counts of CCR2+ puncta. Each dot represents an individual blood vessel. *n* = 49 vessels collected from 4 mice per genotype. ****p < 0.0001 by Student’s t test. (H and I) Representative images and quantification of endothelial CCR2 expression from double immunostaining. Each dot represents an individual animal. *n* = 4–5 per genotype. *p < 0.05 by Student’s t test. (J) Proposed model for the Slc4a4-CCL2-CCR2 axis regulating astrocyte-endothelial interaction. All data are presented as mean ± SEM.

To further elucidate the paracrine signaling molecules sensitive to Slc4a4 loss of function, we collected WT-CM and KO-CM for mass-spectrometry profiling (liquid chromatography-tandem mass spectrometry [LC-MS/MS]) followed by cross-comparison with angiogenesis array analysis (Figure 5A). Our analysis revealed a group of candidate proteins that function in angiogenesis and BBB function (Figure 5B). Since Slc4a4-icKO mice brains showed an increased expression of CD31 (Figure 2A), which is often seen under inflammation conditions,^45^ we further analyzed the chemo/cytokine profile in astrocyte CM. Strikingly, we found an overall elevation of cytokines in KO-CM (Figure 5C). Among those cytokines, CCL2 was particularly interesting as it was also one of the top candidates from LC-MS/MS profiling (Figure 5B). To validate a potential astrocytic Slc4a4-CCL2 cascade, we quantitatively measured CCL2 levels *in vitro* and *in vivo*. ELISA showed that CCL2 was enriched in conditioned media from Slc4a4-depleted astrocytes under both normal (50% increase) and oxygen-glucose deprivation (OGD) conditions (70% increase), the latter mimicking *in vivo* ischemic stroke (Figure 5D). We further measured cortical CCL2 levels, which were upregulated more than 2-fold in Slc4a4-icKO mice under normal and ischemic stroke conditions compared to WT (Figure 5D). Moreover, we found a more than 2-fold increase in astrocytic CCL2 expression in Slc4a4-ablated astrocyte from mouse with either genetic or viral-based deletion at 4 dpi (Figures 5D, 5E, S6A, S6B, and S6C). Existing knowledge suggests that CCL2 promotes endothelial permeability by activating the endothelial CCR2 receptor^46^; therefore, we examined the expression of endothelial CCR2 by RNAscope (2.8-fold increase) and immunostaining (6-fold increase) at the peri-lesion area in Slc4a4-icKO mice brains at 4 dpi (Figures 5F–5I; S6D). In contrast, we did not observe astrocytic CCR2 expression at 4 dpi (Aldh1l1-GFP+; CCR2+, Figure S6A). Together, these data suggest that the Slc4a4-CCL2-CCR2 axis regulates endothelial function in a cell-nonautonomous manner (Figure 5J).

To further demonstrate the putative role of the Slc4a4-CCL2-CCR2 axis in astrocyte-endothelial crosstalk, we perform *in vitro* rescue experiments using an established antibody known to block CCL2 function (FBa)^47,48^ and an established CCR2 antagonist RS504393^49^ (Figure S7). We first treated a mouse endothelial cell line with WT-CM and KO-CM in combination with CCL2 FBa and examined the paracellular pathway by staining for tight-junctional markers ZO-1 and Claudin-5. CCL2 inhibition was found to restore junctional marker expression (Figures S7A–7C). We further found that CCL2 inhibition restored KO-CM-induced paracellular leakage of cultured endothelial cell monolayers by TEER assay (Figure S7D) compared to control levels. Furthermore, we investigated whether the astrocytic Slc4a4-CCL2 axis regulates endothelial transcellular pathways. Western blot analysis of CM-treated endothelia revealed increased pCav-1 expression upon KO-CM treatment, indicating the activation of caveolae-mediated transport, which was rescued by CCL2 inhibition (Figures S7E and S7F). Consistent with our *in vivo* observations that loss of astrocytic Slc4a4 upregulates endothelial CCR2 (Figures 5F–5I), treatment of KO-CM increased endothelial CCR2 expression *in vitro*, which was rescued by CCL2 FBa (Figures S7E and S7F). We further found that KO-CM increased endothelial uptake of albumin compared with WT-CM, which is rescued by CCL2 inhibition (Figures S7G and S7H), confirming that the astrocytic Slc4a4-CCL2 axis regulates caveolae-mediated transcellular transport. Uptake of transferrin (Tf), an indicator for clathrin-mediated endocytosis, was not altered by KO-CM (Figures S7G and S7H). Then, we blocked endothelial CCR2 function using the CCR2 antagonist RS504393 during the incubation with WT- or KO-CM and found that blockage of endothelial CCR2 restored both increased paracellular (ZO-1) and transcellular (pCav-1) pathways in KO-CM-treated endothelia (Figures S7I and S7J). Lastly, we validated the effect of the astrocytic Slc4a4-CCL2 axis on paracellular (ZO-1) and transcellular transport (pCav-1) in primary mouse endothelia (Figures S7K and S7L). Together, these findings indicate that loss of astrocytic Slc4a4 promotes paracellular and transcellular leakage by enhancing the astrocytic CCL2-endothelial CCR2 signaling axis.

### Loss of CCL2 rescues BBB integrity in the absence of Slc4a4 after stroke

**Figure 6.**
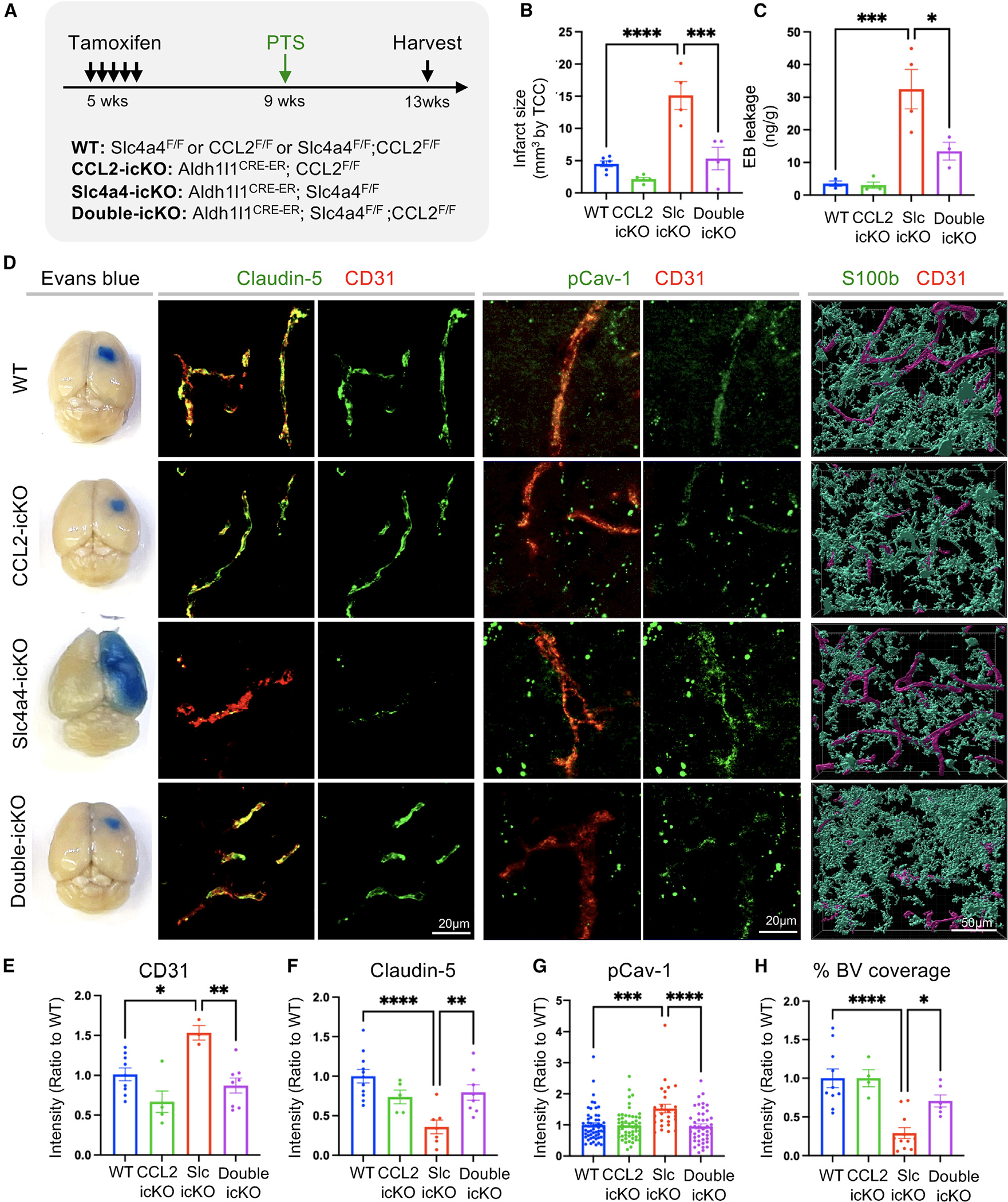
Genetic inhibition of astrocyte-derived CCL2 rescues loss of Slc4a4-induced exacerbated BBB damage after ischemic stroke (A) Experimental scheme of the PTS induction in temporally controlled astrocyte-specific conditional null alleles. Brains were then harvested and analyzed at 4 dpi. (B) Quantification of infarct size based on 2,3,5-triphenyltetrazolium chloride staining of serial 1-mm-thick brain sections at 4 dpi. Each dot represents an individual animal. *n* = 3–6 animals per group. ***p < 0.001, ****p < 0.0001 by two-way ANOVA. (C) Evans blue levels were determined by colorimetric assays. Each dot represents an individual animal. *n* = 3–4 per genotype. *p < 0.05, ***p < 0.001 by two-way ANOVA (D) Representative images of Evans blue leakage, endothelial junctional marker expression (Claudin-5+; CD31^+^), and endothelial pCav-1 at the peri-lesion area. Reactive astrocyte and blood vessel interactions were visualized and reconstructed by double fluorescence staining of S100b and CD31. (E) Quantification of CD31 intensity at the peri-lesion area. Each dot represents each individual animal. *n* = 3–9 animals per group. *p < 0.05, **p < 0.01 by two-way ANOVA. (F) Quantification of Claudin-5 intensity colocalized with CD31 at the peri-lesion area. Each dot represents each individual blood vessel. *n* = 5–16 blood vessels per animal collected from 5–6 animals per group. **p < 0.01, ****p < 0.0001 by two-way ANOVA. (G) Quantification pCav-1 intensity colocalized with CD31 at peri-lesion area. *n* = 28–55 blood vessels collected from *N* = 5–6 animals per group. ***p < 0.001, ****p < 0.0001 by two-way ANOVA. (H) Quantification of blood vessel area covered by astrocytes using IMARIS 3D reconstruction at the peri-lesion area. Each dot represents an individual section. *n* = 4–11 images collected from 4–5 animals per group. *p < 0.05, ****p < 0.0001 by two-way ANOVA. All data are presented as mean ± SEM.

To further determine the putative role of the astrocyte-derived CCL2 in Slc4a4-mediated astrocyte-BBB interaction, we performed double loss-of-function studies in conditional knockout mice for both Slc4a4 and CCL2. As such, we generated temporally controlled conditional null alleles in the astrocytic lineage by crossing floxed alleles with the Aldh1l1-CreER line (Aldh1l1-CreER; Slc4a4^F/F^ denoted as Slc4a4-icKO, Aldh1l1-CreER; CCL2^F/F^ denoted as CCL2-icKO, and Aldh1l1-CreER; Slc4a4^F/F^; CCL2^F/F^ denoted as double-icKO) (Figure 6A). These mice were administered with tamoxifen at 5 weeks of age, and Cre recombination/gene deletions were validated 5 weeks after that (Figure S8A). We first examined BBB integrity by the expression of tight-junctional markers in blood vessels. Notably, deletion of astrocytic CCL2 rescued Claudin-5 loss and blood vessel enlargement caused by the absence of Slc4a4 (Figure S8B), supporting an antagonistic relationship between Slc4a4 and CCL2 in the adult mouse brain under physiological conditions *in vivo*. To examine if the Slc4a4-CCL2 axis is conserved after ischemic stroke, we induced PTS 4 weeks after tamoxifen injection and analyzed the brain at 4 dpi (Figure 6A). After stroke, deletion of astrocytic CCL2 rescued several phenotypes by Slc4a4 loss, including infarct size (Figures 6B and S9A), BBB leakage (Figures 6C and 6D), increased CD31 intensity (Figures 6D and 6E), impaired tight-junctional marker expression (Claudin-5) (Figures 6D and 6F), and enhanced transcellular transport (pCav-1, Cav-1) (Figures 6D, 6G, S9B, and S9C) surrounding the peri-lesion region. Importantly, genetic deletion of the astrocyte-specific CCL2 restored vessel coverage by astrocytes and the number of vessel-associated astrocytes in Slc4a4-icKO mice after stroke (Figures 6D, 6H, S9B, and S9D). These findings provide compelling evidence for the genetic hierarchy in which CCL2 functions downstream of Slc4a4 in BBB maintenance and reconstruction after stroke injury.

Complementary to the genetic rescue experiments, we further evaluated whether pharmacological inhibition of CCL2 rescues exacerbated BBB damage associated with Slc4a4 loss. To this end, we intraperitoneally injected CCL2 FBa at 1 dpi, followed by BBB leakage assessment and marker analysis at 4 dpi (Figure S10A). Notably, CCL2 FBa treatment reversed BBB leakage (Evans blue, fibrinogen) (Figures S10B and S10C), increased CD31 expression (Figures S10B and S10D), and rescued paracellular (Claudin-5) (Figures S10B and S10E) and caveolae-mediated transcellular BBB leakage (pCav-1) (Figures S10B and S10F) in Slc4a4-icKO mice at 4 dpi. Taken together, our results from both genetic and pharmacological approaches support the notion that Slc4a4 governs astrocyte-endothelial interaction by regulating the CCL2-CCR2 pathway under stroke conditions, providing insights into therapeutic implication of CCL2 inhibition in stroke associated with Slc4a4 dysfunction.

### Dysregulation of arginine-NO metabolism upon loss of Slc4a4 contributes to stroke-induced injury

**Figure 7.**
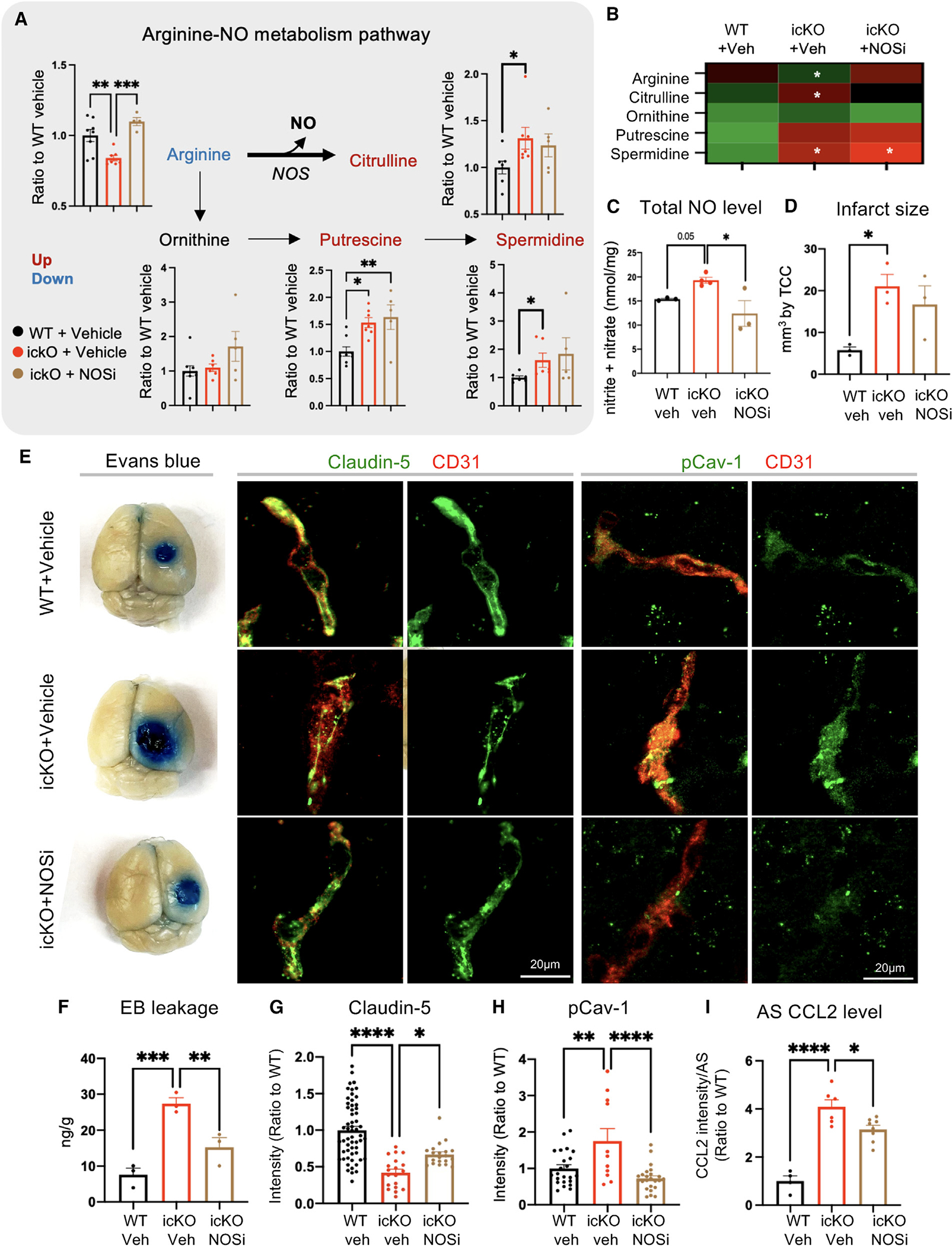
Astrocytic CCL2 dysregulation by the loss of Slc4a4 is partially attributable to arginine metabolism dysregulation (A and B) WT and Slc4a4-icKO mice were intraperitoneally injected with a pan-NOS inhibitor (L-NMMA, 10 mg/kg) from 1 to 3 dpi. Brains were harvested at 4 dpi for analysis of cortical arginine metabolites. Each dot represents an individual animal. *n* = 4–7 animals per group. *p < 0.05, **p < 0.01, ***p < 0.001 by one-way ANOVA. (C) Total NO levels in stroked cortices were measured by the total concentration of nitrite and nitrate using a colorimetric assay. Each dot represents an individual animal. *n* = 3–4 animals per group. *p < 0.05 by one-way ANOVA. (D) Quantification of infarct size based on 2,3,5-triphenyltetrazolium chloride staining of serial 1-mm-thick brain sections from stroked brains at 4 dpi. Data are presented as mean ± SEM. Each dot represents an individual animal. *n* = 3 animals per group. *p < 0.05 by two-way ANOVA. (E) Representative images of Evans blue, Claudin-5 colocalized with CD31, and pCav-1 colocalized with CD31 at the peri-lesion area in the brain. (F) Quantification of Evans blue leakage by colorimetric assay at 4 dpi. Each dot represents an individual animal. *n* = 3 animals per group. **p < 0.01, ***p < 0.010 by two-way ANOVA. (G and H) Quantification of (G) Claudin-5 colocalized with CD31, and (H) pCav-1 colocalized with CD31 at the peri-lesion area at 4 dpi. Each data point represents an individual blood vessel. *n* = 11–56 vessels collected from 3–5 animals per group. *p < 0.05, **p < 0.01, ****p < 0.0001 by one-way ANOVA. (I) *In vivo* astrocytic CCL2 expression after stroke at 4 dpi was quantified from double immunostaining. Each dot represents an individual animal. *n* = 4–7 mice per genotype. *p < 0.05, ****p < 0.0001 by Student’s t test. All data are presented as mean ± SEM.

Given the established link between astrocyte-mediated pH regulation and cellular metabolism, we performed unbiased metabolomic profiling in the cortex of Slc4a4-icKO and WT mice. Arginine metabolism was identified as one of the top altered pathways (Figure S11A). Specifically, we observed increased conversion of arginine to both citrulline and polyamine metabolites, resulting in reduced levels of arginine and increased levels of citrulline, putrescine, and spermidine in Slc4a4-icKO mice compared to WT controls (Figures 7A and 7B). Interestingly, Slc4a4-icKO mice also exhibited an increase in nitric oxide (NO), a byproduct of arginine to citrulline metabolism (Figure 7C), and a factor known to be critical for BBB breakdown as well as promoting pro-inflammatory cytokine production.^50,51^

Given the known role of Slc4a4 in transporting arginine-rich peptides,^52^ we chose arginine metabolism as one of Slc4a4’s downstream pathways to further investigate whether increased citrulline/NO production from arginine metabolism contributes to elevated CCL2 levels and stroke-induced BBB damage in the absence of astrocytic Slc4a4. To this end, we inhibited NO synthetase (NOS), the rate-limiting enzyme of the arginine to citrulline metabolic reaction, by treating Slc4a4-icKO and WT mice with a pan-NOS inhibitor (Figure S11B). As expected, the pan-NOS inhibitor, L-NMMA, rescued excess arginine to citrulline/NO conversion in Slc4a4-icKO brains but not arginine to polyamine conversion (Figures 7A–7C). Although NOS inhibition did not rescue the infarct size, it ameliorated BBB leakage at 4 dpi in Slc4a4-icKO mice (Figures 7D–7F), restored expression of the tight-junction marker Claudin-5, and ameliorated caveolae-mediated transcellular leakage (Figures 7E, 7G, and 7H). Moreover, we observed a partial but significant rescue of CCL2 overproduction in Slc4a4-icKO mice after stroke by an overall NOS inhibition (Figures 7I and S11C). Since inducible NOS (iNOS) is known to produce extra NO under injury, which is known to play a detrimental role in BBB function,^53^ we treated WT and Slc4a4-icKO mice with a selective iNOS inhibitor (1400W, 20 mg/kg) after stroke (Figure S12A). Like pan-NOS inhibition, the iNOS inhibitor restores exacerbated BBB dysfunction and astrocytic CCL2 production in Slc4a4-icKO mice after stroke (Figures S12B–S12F). Together, these results provide an additional mechanism by which loss of Slc4a4 modulates BBB function and astrocytic CCL2 via regulating arginine to citrulline/NO conversion in the context of ischemic stroke.

## DISCUSSION

The mechanism underlying BBB maintenance and recovery after injury remains poorly understood. Our study reveals that astrocytic Slc4a4 loss triggers CCL2 overproduction, exacerbating BBB damage partially through NO-mediated mechanisms. This highlights Slc4a4’s role in regulating astrocyte-endothelial cell interaction, offering a critical regulatory mechanism for BBB recovery after ischemic stroke.

Our results identified Slc4a4’s key role in astrocytogenesis during development and reactive gliosis following ischemic stroke injury. These findings underscore the mechanistic convergence between glia development and regeneration after injury. While such conserved functions have been shown for key astrocytic transcriptional factors,^54^ our study suggests non-transcriptional factors, such as pH, ion homeostasis, and cell metabolism, are also critical for astrocytogenesis. While the process of reactive astrogliosis has been comprehensively characterized at the phenotypic level,^55^ including changes in morphology, metabolism, and inflammatory properties, the molecular connections between reactive astrocyte properties and BBB repair after injury are not fully defined. Emerging evidence has shown a “double-edged sword” property of reactive astrocytes after injury. Some studies have shown that reactive astrocytes can increase endothelial permeability through the secretion of factors such as endothelial growth factor and interleukin-6.^56–58^ Consistent with these findings, our study demonstrates that the loss of Slc4a4 leads to increased secretion of the pro-inflammatory factor CCL2, resulting in enhanced BBB permeability. Conversely, other studies have indicated that the ablation or impairment of reactive gliosis can exacerbate BBB damage after injury.^59–61^ In line with these observations, our study reveals that the loss of Slc4a4 reduces the overall number of reactive astrocytes, which is associated with exacerbated stroke-induced damage. Collectively, our study defines a mechanism through which Slc4a4 affects BBB integrity by regulating reactive astrogliosis after stroke.

Pathological pH decline is often observed after ischemic stroke, partially owing to hypoxia, which then triggers a sharp response by ion transporters across multiple cell types.^18^ Recent studies showed that Slc4a4 is activated and releases HCO_3_^−^ to neutralize rapid acidification from synaptic transmission.^25^ It is reported that loss of astrocytic Na^+^/H^+^ exchanger isoform 1 (NHE1) reduced astrogliosis and ameliorated BBB damage.^62^ The functional difference between Slc4a4 and NHE1 should be noted. While NHE1 is an H^+^ extrusion in astrocytes regardless of the extracellular pH or ion concentration,^63^ Slc4a4 transports sodium/bicarbonate bidirectionally across the cell membrane depending on the electrogenic gradients,^64,65^ making it a versatile “modulator” for drug targeting in brain disorders.

Injury-induced BBB leakage has been linked with increased paracellular transport regulated by tight-junction proteins and elevated caveolae-mediated transcellular transport.^66^ Mounting evidence suggests that the CCL2-CCR2 signaling is detrimental to ischemic stroke recovery and BBB function.^67,68^ Increased CCL2 leads to loss of endothelial tight-junction marker expression^69^ and upregulation of caveolae. These findings, in conjunction with our own results, imply that an excess of CCL2 impairs BBB function under pathological conditions. While our study found very low expression of CCR2 mRNA and protein in normal or stroked WT brain, consistent with previous endothelial transcriptome atlases,^70,71^ we found that loss of Slc4a4 results in an upregulation of endothelial CCR2, contributing to exacerbated BBB damage after stroke. Our study revealed Slc4a4 as a key regulator for astrocyte-endothelial interaction via the CCL2-CCR2 axis.

Several types of cells in the brain can produce NO. On the one hand, endothelial cells utilize endothelial nitric oxide synthase (eNOS) to produce NO at physiological states to maintain normal endothelial function.^72^ On the other, several other types of cells, including astrocytes and microglia, utilize iNOS to produce extra harmful NO to impair BBB under injury.^53^ While our study shows that loss of astrocytic Slc4a4 stimulates NO production, further studies will examine the expression and activity of key enzymes involved in brain arginine metabolism to define the Slc4a4-arginine/NO relationship in a cell-type-specific manner.

### Limitations of the study

Due to technical limitations regarding the manipulation of intracellular or extracellular pH *in vivo*, one remaining question from our study concerns whether pH directly regulates astrocyte development and reactivity. Additionally, considering that astrocyte interaction with blood vessels may depend on physiological factors such as blood flow and respiratory activity, another limitation of our study is the utilization of post-mortem brain tissues to assess blood vessel complexity and its interaction with astrocytes. This approach may not fully reflect the real-time, *in vivo* physiological changes.

## STAR★METHODS

### RESOURCE AVAILABILITY

#### Lead contact

Further information and requests for resources and reagents should be directed to and will be fulfilled by the lead contact, Hyun Kyoung Lee (hyunkyol@bcm.edu).

#### Materials availability

Unique resources and reagents generated in this study are available from the lead contact with a completed Material Transfer Agreement.

#### Data and code availability

- Microcopy data reported in this paper will be shared by the lead contact upon request.
- This paper does not report original code.
- Any additional information required to reanalyze the data reported in this paper is available from the lead contact upon request.

### EXPERIMENTAL MODEL AND STUDY PARTICIPANT DETAILS

#### Animals

All mice were maintained and studied according to protocols approved by the Institutional Animal Care and Use Committee of Baylor College of Medicine. All genotypes were on the C57BL/6J strain and blinded for data acquisition and analysis, and experiments included both genders and age-matched controls. Aldh1l1-EGFP transgenic mice were used for astrocyte-specific labeling.^76^ For temporal expression of Cre in astrocytes, Aldh1l1-Cre^ERT2^ (The Jackson Laboratory, stock number 029655) was used. Slc4a4-floxed mice were generated and provided by Dr. Shull.^74^ CCL2-floxed animals were purchased from The Jackson Laboratory (stock number 029655). To induce gene deletion during development, animals were injected subcutaneously with tamoxifen (Sigma T5648, 100 mg/kg, dissolved in corn oil) at postnatal day 3 (P3). To induce gene deletion in adult mice, animals were injected intraperitoneally with tamoxifen (100 mg/kg) daily for 5 consecutive days from 5 to 6 weeks old. For AAV virus-mediated deletion of Slc4a4, AAV-PHP.eB-GfaABC_1_D-Cre virus (Addgene #196410) or control AAV-PHP.eB-GfaABC_1_D-tdTomato (Addgene #44332) virus was retro-orbitally injected in Slc4a4 F/F mice at a dose of 1 × 10^11^ vg/mouse at 2 weeks before stroke induction.

### METHOD DETAILS

#### Photothrombotic focal ischemia model

Focal ischemic sites were generated in the cortex of 10 to 12-week-old mice. Briefly, mice were anesthetized using isoflurane, and an incision was made to expose the skull. A customized dark PVC mask with a 2 mm diameter aperture was placed over the exposed skull to limit the laser illumination to a location 1 mm anterior and 1 mm lateral to bregma. Then, mice were injected intravenously with 20 mg/kg of Rose Bengal dye (Sigma 330000, 10 mg/mL in sterile saline), followed by immediate exposure to light from Leica KL 300 LED for 15 min. The surgical site was then sutured, and the animals were allowed to recover in sternal recumbency until fully awake.

#### Quantification of stroke infarct size

The size of the stroke lesion was evaluated by TTC staining or H&E staining using serial brain sections. For TTC staining, stroked brains were removed and sectioned into 1mm-thick brain slides using a brain matrix (Ted Pella) within 3 min of harvest. Brain slices were then immersed in a 2% solution of TTC in normal saline at 37°C for 30 min, after which sections were fixed in 4% PFA for photography. For H&E staining, hematoxylin was stained for 5 min, then rinsed with tap water until no more color changes. 2–3 dips in the acidic solution (1% HCl in 70% EtOH) reduced the background. After rinsing with tap water, eosin was stained for 1 min, followed by tap water rinsing. Finally, dehydration was accomplished by three 5-min incubations with 95% EtOH, 100% EtOH, and Xylene. Stained slides were mounted with Permount mounting medium. Images were obtained using a Nikon spinning disc microscope.

#### Drug administration

For the pharmacological CCL2 rescue experiment, mice were intraperitoneally injected with CCL2 functional blocking antibody (R&D; AF-479-NA, 0.5 mg/kg) at 1 dpi; normal goat IgG (R&D; AB-108-C) was used as control. For the inhibition of total NOS, mice were intraperitoneally injected with a pan-NOS inhibitor L-NMMA (Sigma M7033, 10 mg/kg) once daily from 1 to 3 dpi; saline was used as a control. For the selective inhibition of iNOS, mice were intraperitoneally injected with an iNOS inhibitor 1400W (Abcam, 20 mg/kg) once daily from 1 to 3 dpi; saline was used as a control. Upon harvesting of the tissue at designated time points, the animals were perfused, and tissues were processed as described below.

#### BrdU labeling *in vitro* and *in vivo*

To evaluate endothelial cell proliferation *in vitro* after conditioned medium treatment, 10μm of BrdU was added to cultured cells 6 h before collecting. To evaluate astrocyte proliferation *in vivo* after stroke, we administered multiple doses of BrdU (200 mg/kg, every 12 h from 1 to 3 dpi) before harvesting. Brains were harvested and processed as described above. For the BrdU staining, an additional 2N HCl treatment for 30 min was performed.

#### Immunohistochemistry

Unless otherwise specified, deeply anesthetized animals were transcardially perfused with 4% paraformaldehyde (PFA)/PBS solution. Fixed brains were extracted, followed by overnight PFA fixation and gradient sucrose dehydration. Brains were embedded in OCT (Tissue TEK) and sectioned with a cryostat (Leica) at a thickness of 40μm (free floating section) or 15μm (attached section). For immunofluorescence staining of tight junction makers (ZO-1, Claudin-5, VE-Cadherin), transcellular markers (Caveolin-1, pCaveolin-1), and CCL2, animals were perfused with PBS solution. Brains were extracted, immersed in OCT, and snap-frozen. Brains were sectioned at a thickness of 15μm and fixed in 95% ethanol before staining. Tissue sections were washed in PBS, permeabilized with PBST (0.3% Triton in PBS), blocked with 10% normal goat serum in PBST, and then incubated with primary antibodies overnight at 4°C, followed by secondary antibody and nuclear staining with DAPI. Stained slides were mounted with Vectashield Antifade mounting medium. Images were obtained by Zeiss Imager.M2m equipped with ApoTome.2 or Leica STED microscope system.

#### FAC-sorted astrocytes for RT-qPCR

Cortex was microdissected and dissociated into a single-cell suspension in FACS buffer (Leibowitz Media, supplemented with 1 μg/mL BSA, 10 mM HEPES pH 7.05, pen/strep, and 25 μg/mL DNase I). For cell sorting, a BD FACSAria III instrument (100-μm nozzle and 20-p.s.i. setting) was used with FACSDIVA software. Around 100,000 GFP+ astrocytes were collected directly into 500 μL of Buffer RLT (QIAGEN 79216) with 1% β-Mercaptoethanol. Finally, each sample was vortexed and rapidly frozen on dry ice. RNA was extracted from pelleted cells using RNeasy Micro Kit (QIAGEN 74004), followed by cDNA synthesis (AmfiSure ONE PCR Master Mix P7000–050) and RT-qPCR (AmfiRivert cDNA Synthesis Platinum Master Mix, R5600) on a BioRad CFX Duet Real-Time PCR System. qPCR was carried out at 95°C for 30 s, 40 cycles of 95°C for 5 s, and 60°C for 30 s, with subsequent melting curve analysis. Expression of transcripts of target genes was normalized to Gapdh. Primer sequence used for mouse Slc4a4 was obtained from published literature.^77^

#### *In situ* hybridization and RNAscope

PFA-fixed brain sections were acquired as described above. For *in situ* hybridization, RNA probes (mouse *Slc4a4* cDNA sequence) with DIG and/or FITC labels were generated in-house to detect the *Slc4a4* RNAs. All probes were tested for specificity, and sense probes were included in the experiments as a control. Detailed procedures and reagents were described before.^78^ For RNAscope, tissue sections were stained using the RNAscope Multiplex Fluorescent Reagent Kit v2 (Advanced Cell Diagnostics, 323100) in combination with immunofluorescence integrated co-detection kit (Advanced Cell Diagnostics, 323180) following manufacturer’s instruction. Brain tissues were incubated with primary antibody against endothelial marker CD31 prior to mouse *CCR2* probe (Advanced Cell Diagnostics, S01681). CCR2+ puncta were counted using Fiji for *CCR2* mRNA expression.

#### 3D image and lightsheet confocal

To label blood vessels *in vivo*, tomato-labeled lectin (Lycopersicon Esculentum) (VECTOR Laboratories, #DL-1178–1) was injected into mice via tail vein (50 μL) and intracardially (100 μL) before harvesting. Astrocytes were genetically labeled with Aldh1l1-GFP. Mice were then perfused, and brains were fixed as described above. As described previously,^79^ brains were then cleared using the X-CLARITY platform (Logos Biosciences), followed by imaging using Lightsheet Z.1 microscope (Zeiss).

#### Astrocyte morphology and astrocyte-blood vessel coverage analysis

For all morphological analysis by 3D rendering, astrocytes (Aldhl1l-GFP) or blood vessels (lectin) were randomly picked and analyzed by a person blinded to the experimental groups (H.O.) All the images were obtained using constant exposure settings across all groups. For astrocyte volume analysis, late P0/early P1 pups were sedated by hypothermia until anesthetized.1μL of AAV-PhP.eB-GfaABC1D-mCherry-CAAX construct (titer~1 × 10^12^ GC/mL) mixed with Fast Green Dye was injected into the lateral ventricle of one hemisphere using a pulled glass pipette. To assess the volume of individual astrocytes in the L 4–5 visual cortex, 100 μm-thick floating sections containing astrocytes labeled sparsely with mCherry-CAAX were used. High magnification images containing an entire astrocyte (60–80 μm z stack) were acquired by Zeiss Imager.M2m equipped with ApoTome.2 with the 63x objective. Astrocytes in which the entire astrocyte could not be captured within the section were excluded. Astrocyte volume was calculated using Imaris Bitplane software with the surface tool.

#### Calcium imaging

Calcium imaging of cortical astrocytes was performed as previously described.^80^ Briefly, AAV2/9-GfaABC_1_D-GCaMP6f virus (Addgene #52925) was stereotaxically injected into the cortex 2 weeks before imaging. On the day of imaging, animals were anesthetized with isoflurane, followed by intracardial injection of tomato-labeled lectin (Lycopersicon Esculentum) (VECTOR Laboratories, #DL-1178–1) to label blood vessels. The brain was isolated and sliced quickly. Calcium activity was collected at 1 Hz for 200s using a two-photon resonant microscope (LSM 7MP, Zeiss) with a Coherent Chameleon Ultra (II) Ti-sapphire laser tuned to 900 nm. Relative changes in fluorescence of soma, primary branches, and endfeet were quantified using GECIquant with ImageJ. MATLAB software was used to quantify amplitude and frequency.

#### BBB leakage assessment *in vivo*

BBB leakage *in vivo* was assessed by extraversion assays of Evans blue (Sigma), 3 kDa dextran, and EZ-Link Sulfo-NHS-Biotin (MW = 443 kDa, Thermo Fisher Scientific) as described below.

For Evans blue, mice were injected intraperitoneally with a filtered 2% Evans blue solution in PBS (15 mL/kg), and brains were harvested 24 h (non-injured animal) or 6 h (stroke animal) after injection. Brains were weighed and incubated with formamide (2 v/w) for 24 h at 65°C. Subsequently, the samples were briefly centrifuged, and supernatants were taken to measure absorbance at 600 nm with a standard curve of Evans blue dye. The final concentration of Evans blue in the tissue was calculated as ng/g tissue.

3 kDa dextran leakage into the brain was assessed as previously described. Mice were intravenously injected with 3 kDa A488-dextran (10 mg/kg, ThermoFisher Scientific D3306). After 2 h, mice were anesthetized and perfused with ice-cold PBS (pH 7.4) and PFA. Brains were harvested, PFA-fixed, dehydrated, sectioned, and imaged as described above. The leakage of injected dextran into brain parenchyma was measured based on intensity and quantified using ImageJ software, as described in the image analysis section.

Small molecule leakage into the brain was assessed by EZ-Link Sulfo-NHS-Biotin. Briefly, mice were intracardially injected with 200 μL biotin (20 mg/mL) right before harvesting. Brains were snap-frozen, followed by sectioning and ethanol fixation, as described above. Sections were stained with Alexa 535-streptoavidin (Invitrogen, 1:1,000), mounted, and imaged with a Nikon spinning disc microscope. The leakage of injected biotin into brain parenchyma was quantified using ImageJ software, as described in the image analysis section.

#### Primary astrocyte culture and conditioned media collection

Astrocytes were obtained from the cortices of P0 to P1 WT and Slc4a4-icKO (Slc4a4^F/F^; Aldh1l1-cre) mice and cultured in DMEM/F12 medium containing 10% FBS and 1% penicillin-streptomycin. Cells were then plated into poly-D-lysine-coated T75 culture flasks, and media were changed every 3–4 days. After 7 days, flasks were placed on an orbital shaker at 120 rpm overnight to remove microglia and oligodendrocyte precursors.

For conditional media collection for cytokine array and mass-spec profiling, astrocytes were subcultured into 12-well plates and allowed to grow until confluent. 24 h before collection, the medium was switched to a medium free of phenol and FBS. For the oxygen-glucose deprivation (OGD) model, the prepared cells were cultured in glucose-free Dulbecco modified Eagle medium (GenDEPOT) in a 37°C incubator containing a humidified gas mixture of 1% O_2_/95% N_2_/4%CO_2_ for 2 h. For reoxygenation, the cells were incubated for 24 h with normal DMEM containing 1% FBS in a 37°C incubator containing humidified 5% CO_2_/95% air.

#### *In vitro* BBB leakage assay

For *in vitro* TEER assay, astrocytes were seeded at the bottom of the transwell plate (0.4-μm pore size, 12-well; Corning) at a density of 3 × 10^5^ cells per filter. After allowing the astrocytes to grow for 48 h, immortalized mouse endothelial bEnd3 cells (American Type Culture Collection) were plated in the inserts (5 × 10^4^ cells per well). TEER measurements were then made 24 h after bEnd3 seeding using an EVOM resistance meter (World Precision Instruments) with Stx2 electrodes. TEER resistance was measured in ohms after subtracting resistance from blank transwell inserts.

For *in vitro* endocytosis assay, bEnd3 cells were incubated with 50 μg/mL Alexa 488-transferrin (ThermoFisher Scientific, T13342) or Texas Red-albumin (ThermoFisher Scientific, A13100) for 30 min at 37°C, 5% CO_2_, as described previously.^81^ Cells were then rinsed three times with ice-cold PBS, fixed with 4% paraformaldehyde (PFA) and stained with DAPI. The samples were imaged and analyzed with Zeiss Imager.M2m equipped with ApoTome.2.

#### Western blot

Cells were lysed with lysis buffer (150 mM NaCl, 50 mM Tris–HCl, 1 mM EDTA, 1% Triton X-100, 1% NP-40) containing 1X protease inhibitor cocktail (GenDEPOT, P3100) and electrophoresed on 10% SDS-PAGE (10 μg protein) and transferred to nitrocellulose membranes. After blocking with 5% nonfat milk, the membranes were incubated with primary antibodies and rabbit anti-Gapdh as a loading control. Blots were incubated with HRP-conjugated secondary antibodies. Immunoreactivity was developed using an ECL reagent (GenDEPOT, W3653) and imaged with Bio-Rad Gel Imager ChemiDoc.

#### Extracellular pH after ischemic stroke

Mice with ischemic stroke were intraperitoneally injected with 100 μL pHLIP-ICG probe (1 mg/kg, synthesized and provided by Y.K.R.). 24 h after injection, brains were harvested and imaged using the Bruker Xtreme Imager with 735 nm excitation and 830 nm emission wavelength. The fluorescence intensities in regions of interest were calculated using ImageJ.

#### Cytokine array

Astrocyte-conditioned media was collected from 3 different mice, pooled, and centrifuged for 10 min at 1,000 rpm. Protein concentrations were determined by Bradford assay and adjusted to the same concentration. Conditioned media was then measured in duplicates with Mouse Cytokine Array Panel A (ARY006, R&D; Systems) according to the manufacturer’s instructions. Signal detection was performed using a ChemiDoc Imaging System (Bio-Rad, Hercules, CA, USA), and the signal was quantified using Image Lab software (Bio-Rad).

#### Mass-spec protein profiling

With an established method,^80,82^ conditioned medium was mixed with lysis buffer (50 mM ammonium bicarbonate, 1 mM CaCl_2_) and boiled at 95°C for 3 min. All denaturing procedures were repeated two times. Peptide supernatant was obtained by digesting 20 mg of proteins with trypsin (T9600, GenDEPOT), followed by extraction with 50% acetonitrile and 2% formic acid. Vacuum-dried peptides were dissolved in 10 mM ammonium bicarbonate buffer, pH 10 adjusted by NH4OH, and were subjected to reverse phase column chromatography with a micropipette tip C18 column, followed by fractionation with stepwise acetonitrile gradient into 15 elution groups. Eluent was pooled into five pools and vacuum-dried for nano HPLC-MS/MS. Peptides were resuspended in the loading solution (5% methanol containing 0.1% formic acid) and subjected to analysis with a nano LC 1000 coupled with Orbitrap FusionTM mass spectrometer (Thermo Fisher Scientific). Data were analyzed with proteome discoverer 2.1 interface (Thermo Fisher Scientific), and detected peptides were assigned into gene products by PyGrouper and Tackle analysis platform from iSpec.

#### CCL2 measurement

Fresh brain cortical tissue was used, and stroked tissue was microdissected based on a white, opaque, scar-like appearance. CCL2 concentration was measured by Mouse CCL2/JE/MCP-1 Quantikine ELISA Kit (Bio-Techne; MJE00B) according to the manufacturer’s instructions.

#### Metabolomics analysis

Mouse cortices were flash-frozen in liquid nitrogen and stored at −80°C until analysis. Prior to liquid chromatography analysis, samples were placed on ice and suspended with methanol:acetonitrile:water (5:3:2, v to v) to a concentration of 30 mg/mL. Glass beads (GB10, Next Advance) were added to each tube and placed into a Bullet Blender (Next Advance) at setting 3 for 5 min at 4°C to homogenize tissue. Suspensions were then vortexed continuously for 30 min at 4°C. Insoluble material was removed by centrifugation at 10,000 × *g* for 10 min at 4°C and supernatants were collected. LC-MS analysis was conducted on randomized samples using the Vanquish UHPLC system linked to a Q Exactive Orbitrap mass spectrometer (Thermo Fisher Scientific) in both positive and negative ion modes. The LC system is equipped with a Kinetex C18 column (150 mm × 2.1 mm, 1.7 mm; Phenomenex) with mobile phases and 5-min gradients.^83^ Mobile phases for positive ion mode consist of phase A (water with 0.1% formic acid) and phase B (acetonitrile with 0.1% formic acid). Mobile phases for negative ion mode consist of phase A (5% acetonitrile, 95% water, 1 mM ammonium acetate) and phase B (95% acetonitrile, 5% water, 0.5 mM ammonium acetate). Acquired spectra (.raw) were converted to.mzXML format using RawConverter, and signals were annotated and integrated using Maven (Princeton University) with the KEGG database. Metabolite assignments and isotopologue distributions were analyzed using Maven (Princeton), while metabolic pathway analysis utilized the MetaboAnalyst 5.0 package.

#### NO measurement

Fresh brain cortical tissue was used, and stroked tissue was microdissected based on a white, opaque, scar-like appearance. NO concentration was measured by Griess methods (Nitrate/Nitrite Colorimetric Assay Kit; Cayman Chemical Co; 780001) according to the manufacturer’s instructions.

### QUANTIFICATION AND STATISTICAL ANALYSIS

#### Image quantification

For image analysis in stroke brains, the lesion border is identified based on the autofluorescence signal and brightfield tissue histology, and the peri-lesion is defined as 150 μm outside the border. All the image analyses were performed by the person (H.O. and T.C.) who were blinded to the experimental groups.

All the images were obtained using constant exposure settings across all groups within the same marker analysis. For intensity quantification, the region of interest (ROI) was outlined. For co-expression analysis, fluorescence images were split into single-channel images, and ROI was outlined based on marker staining (e.g., CD31 for endothelia, GFAP, or Aldh1l1 for astrocytes). Then the same ROI will be applied to the co-stained channel (e.g., claudin-5, caveolin-1, phospho-caveolin, CCL2, and CCR2) to quantify the mean density. The background of each image was measured in an area without fluorescence signal within each section and subtracted from the mean density of the target protein measured above. 2–3 sections were analyzed per animal and averaged as one biological data point unless otherwise specified in the figure legends. Lastly, the data presented were normalized to the WT-control average.

For glial scar quantification, the glial scar is defined by a dense limiting GFAP+ border consisting of fibrous reactive astrocytes surrounding the core lesion of stroke, as shown in Figure S3F. The thickness of the glial scar is measured as the maximum distance between the dorsal and the ventral side of the glial scar.

Morphological Sholl analysis of individual astrocytes was performed using the Filament module in the IMARIS software 10.3.0 (Oxford Instruments). Maximum orthogonal projections of z stack images (1 μm interval, 40–80 slides) were imported to IMARIS, and astrocyte branches were outlined using the Filament Autopath algorithm with a dendrite starting point diameter of 5.00 μm and a dendrite seed point diameter of 0.216 μm. Following automated tracing, filament structures were then manually pruned to eliminate false positive branches.

For astrocyte-blood vessel 3D rendering, the fluorescence signal was reconstructed with the surface tool, and the area of a blood vessel (lectin) covered by astrocytes (Aldh1l1-GFP) was calculated by the IMARIS software.

#### Statistics

GraphPad Prism v9 was used to generate graphs and statistical analysis. Data were reported as mean ± SEM. Significance was calculated using two-tailed, unpaired Student’s t-tests for two-group comparisons, or two-way ANOVAs for four-group comparisons, followed by Sidak’s post hoc analysis. *p < 0.05, **p < 0.01, P*** <0.001, and ****p < 0.0001 were considered to indicate statistical significance. Sample size for each experiment is indicated in the corresponding figure legend. Number of biological replicates, either animals (*N*) and/or cells/images/blood vessels (*n*), used for quantifications of each experiment were indicated in the respective figure legends.

## Supplementary Material

1

2

## Figures and Tables

**KEY RESOURCES TABLE:** 

REAGENT or RESOURCE	SOURCE	IDENTIFIER

Antibodies		

GFAP	Agilent	Z0334; RRID: AB_10013382
NeuN	Millipore	MAP377; RRID: AB_2298772
GFP	Chromotek	PABG1-100; RRID: AB_2749857
BrdU	Abcam	Ab6326; RRID: AB_305426
S100b	Agilent	Z0311; RRID: AB_10013383
CD31	BD Bioscience	550274; RRID: AB_393571
Glut1	Millipore	07-1401; RRID: AB_1587074
AQP4	Sigma	A5971; RRID: AB_258270
Zo-1	Invitrogen	40-2200; RRID: AB_2533456
Alexa 488 conjugated Claudin-5	Invitrogen	3532588; RRID: AB_2532189
Mouse Albumin Polyclonal Antibody, FITC	Thermo Fisher	A90-234F; RRID: AB_67126
BD Pharmingen^™^ Purified Rat Anti-Mouse CD144 (VE-Cadherin	BD Biosciences	555289; RRID:AB_395707
CCL2	PeproTech	500-P113; RRID: AB_147738
CCL2	R&D	AF-479; RRID: AB_354500
CCR2	Abcam	Ab273050; RRID: AB_2893307
Cav-1	BD Bioscience	610406; RRID: AB_2314110
pCav-1	BD Bioscience	611339; RRID: AB_398863
Gapdh	GeneTex	GTX100118; RRID: AB_1080976
Goat anti-Mouse IgG1, Alexa Fluor^™^ 568	Invitrogen	A21124; RRID: AB_141611
Goat anti-Mouse IgG1, Alexa Fluor 488	Invitrogen	A21121; RRID: AB_2535764
Goat anti-Rat IgG (H + L), Alexa Fluor^™^ 647	Invitrogen	A48265; RRID: AB_2895299
Goat anti-Rat IgG (H + L), Alexa Fluor^™^ 568	Invitrogen	A11077; RRID: AB_2534121
Goat anti-Rabbit IgG (H + L), Alexa Fluor 488	Invitrogen	A11034; RRID: AB_2576217
Goat anti-Rabbit IgG (H + L), Alexa Fluor 568	Invitrogen	A11036; RRID: AB_10563566
Goat anti-Mouse IgG (H + L), Alexa Fluor^®^ 488	Invitrogen	A11001; RRID: AB_2534069
Goat anti-Mouse IgG (H + L), Alexa Fluor^®^ 568	Invitrogen	A-11057; RRID: AB_2534104

Bacterial and virus strains		

AAV2/9-GfaABC_1_ D-GCaMP6f	Addgene	#52925
AAV-PhP.eB-GfaABC_1_ D-mCherry-CAAX	Stogsdil et al.^34^	N/A
AAV-PhP.eB-GfaABC_1_ D-TdTomato	Addgene	#44332
AAV-PhP.eB-GfaABC_1_ D-Cre	Addgene	#196410

Chemicals, peptides, and recombinant proteins		

Tamoxifen	Sigma	T5648
Rose Bengal dye	Sigma	330000
CCR2 antagonist	Tocris	2517/10
L-NMMA	Sigma	M7033
1400W	Abcam	ab120165
BrdU	Sigma	19-160
Lycopersicon Esculentum	VECTOR Laboratories	#DL-1178-1
Evans blue	Sigma	E2129
Dextran, Fluorescein, 3000 MW, Anionic, Lysine Fixable	Thermo Fisher	D3306
EZ-Link Sulfo-NHS-Biotin	Thermo Fisher	PI21335
pHLIP-ICG probe	Crawford et al.^73^	N/A

Critical commercial assays		

Mouse Cytokine Array Panel	R&D Systems	ARY006
Mouse CCL2/JE/MCP-1 Quantikine ELISA Kit	Bio-Techne	MJE00B
Nitrate/Nitrite Colorimetric Assay Kit	Cayman Chemical Co	780001

Experimental models: Cell lines		

bEnd3 cells	American Type Culture Collection	CRL-2299; RRID: CVCL0170
C57BL/6 Mouse Primary Brain Microvascular Endothelial Cells	Cellbiologics	C57-6023

Experimental models: Organisms/strains		

Aldh1l1-CreER; Slc4a4^F/F^; Aldh1L1-EGFP	This paper	N/A
Slc4a4^F/F^; Aldh1L1-EGFP	This paper	N/A
Aldh1l1-CreER; Slc4a4^F/F^	This paper	N/A
Slc4a4^F/F^	Vairamani K et al.^74^	N/A
CCL2^F/F^	The Jackson Laboratory	029655
Aldh1l1-CreER; CCL2^F/F^	This paper	N/A
Aldh1l1-CreER; Slc4a4^F/F^; CCL2^F/F^	This paper	N/A
Slc4a4^F/F^; CCL2^F/F^	This paper	N/A

Oligonucleotides		

Genotyping Slc4a4 Forward: TGGTGGCTTAAATTGCAAATGGC	Sigma	N/A
Genotyping Slc4a4 Reverse: CATAACCCACTAAGTCCAGTACG	Sigma	N/A
Genotyping Cre Forward: CACCATTGCCCCTGlIlCACTATC	Sigma	N/A
Genotyping Cre Reverse: GTGTTGCCGCGCCATCTGC	Sigma	N/A
Genotyping CCL2 mutant Forward: CAGAGGCTGAAGCTGAAGGA	Sigma	N/A
Genotyping CCL2 wild type Forward: ACTGCATCTGCCCTAAGGTCT	Sigma	N/A
Genotyping CCL2 Reverse: AGGCATCACAGTCCGAGTCA	Sigma	N/A
Genotyping Aldh1l1-EGFP Forward: CCTCTGGCTGCTCCTTCAACAG	Sigma	N/A
Genotyping Aldh1l1-EGFP Reverse: GGTCGGGGTAGCGGCTGAA	Sigma	N/A

Software and algorithms		

Zen microscopy software	Zeiss	RRID:SCR_013672
Prism 9	GraphPad	RRID: SCR_002798
Fiji	NIH	RRID: SCR_022512
IMARIS software 9.3.0	Bitplane	RRID:SCR_007370
MetaboAnalyst 5.0 package	Xia et al.^75^	RRID:SCR_015539
Fiji	NIH	RRID: SCR_022512
MATLAB	MathWorks	RRID:SCR_001622

## Highlights

- Astrocytic Slc4a4 is required for proper astrocyte development and reactive astrogenesis
- Astrocytic Slc4a4 is important for BBB function under healthy and stroke conditions
- Astrocytic Slc4a4-CCL2 and endothelial CCR2 axis controls BBB repair after stroke
- Slc4a4 deletion dysregulates arginine-NO metabolism after stroke
