# Supplementary material for: Astrocytic Slc4a4 regulates blood-brain barrier integrity in healthy and stroke brains via a CCL2-CCR2 pathway and NO dysregulation
Source: Cell Rep. Author manuscript; Available in PMC 2024 Jun 27. (PMC11210630; doi:10.1016/j.celrep.2024.114193)
Supplement: 1 [file NIHMS2000725-supplement-1.pdf]

Cell Reports, Volume *43*

Supplemental information

Astrocytic Slc4a4 regulates blood-brain barrier integrity in healthy and stroke brains via a CCL2-CCR2 pathway and NO dysregulation

Qi Ye, Juyeon Jo, Chih-Yen Wang, Heavin Oh, Jiangshan Zhan, Tiffany J. Choy, Kyoung In Kim, Angelo D'Alessandro, Yana K. Reshetnyak, Sung Yun Jung, Zheng Chen, Sean P. Marrelli, and Hyun Kyoung Lee

Figure S1 Related to Figure 1

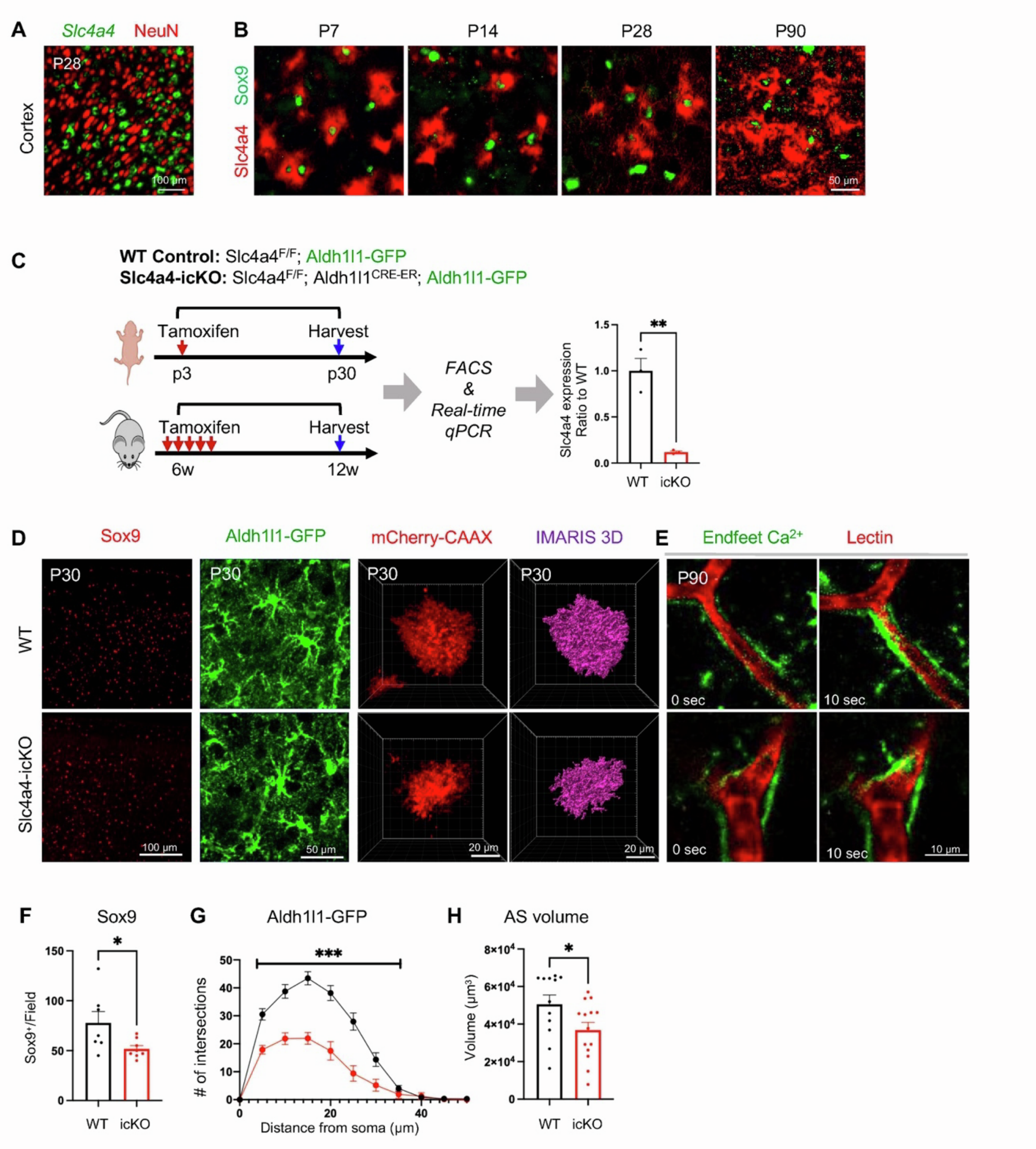

Figure S1. Loss of astrocytic Slc4a4 impairs astrocyte development but does not not change endfeet Ca^{2+} activity in adult mice. (A) Double in situ-immunofluorescence staining of Slc4a4 in neurons (NeuN) in P28 mouse cortex. **(B)** Double in situ-immunofluorescence staining of Slc4a4 in astrocyte lineage (Sox9) in developmental and adult mouse cortex. **(C)** Schematic of the generation of astrocyte-specific Slc4a4 knockout mice with Aldh1l1-GFP reporter. Deletion of Slc4a4 was confirmed by quantitative RT-PCR of FAC-sorted GFP+ astrocytes. Data are presented as mean ± SEM. n = 3 animals per genotype. *p<0.05 by Student's t-test. **(D)** Immunofluorescence staining of astrocyte markers (Sox9, Aldh1l1-GFP) in the cortex from WT and Slc4a4-icKO mice at P30. Astrocyte morphology is labeled at single-cell resolution using AAV-

PhP.eB-GfaABC$_1$D-mCherry-CAAX by intracerebral injection at P1, followed by IMARIS 3D reconstruction. **(E)** Representative images of astrocytic endfeet spontaneous calcium activity from WT and Slc4a4-icKO mice at P30. **(F)** Quantification of the number of Sox9+ cells from WT and Slc4a4-icKO cortices at P30. Data are presented as mean ± SEM. Each dot indicates an individual animal. N= 7 animals per group. *$p<0.05$ by Student's t-test. **(G)** Overall complexity of astrocytes (Aldh1l1-GFP) was measured by Sholl analysis. Data are presented as mean ± SEM. n = 6-8 cells collected from 4-6 mice per genotype. ***$p<0.001$ by two-way ANOVA. **(H)** Astrocyte volume was reconstructed and quantified using IMARIS software. Each dot represents an individual astrocyte. n = 14-18 astrocytes collected from 4 mice per group. Data are presented as mean ± SEM. *$p<0.05$ by Student's t-test.

Figure S2 Related to Figure 2

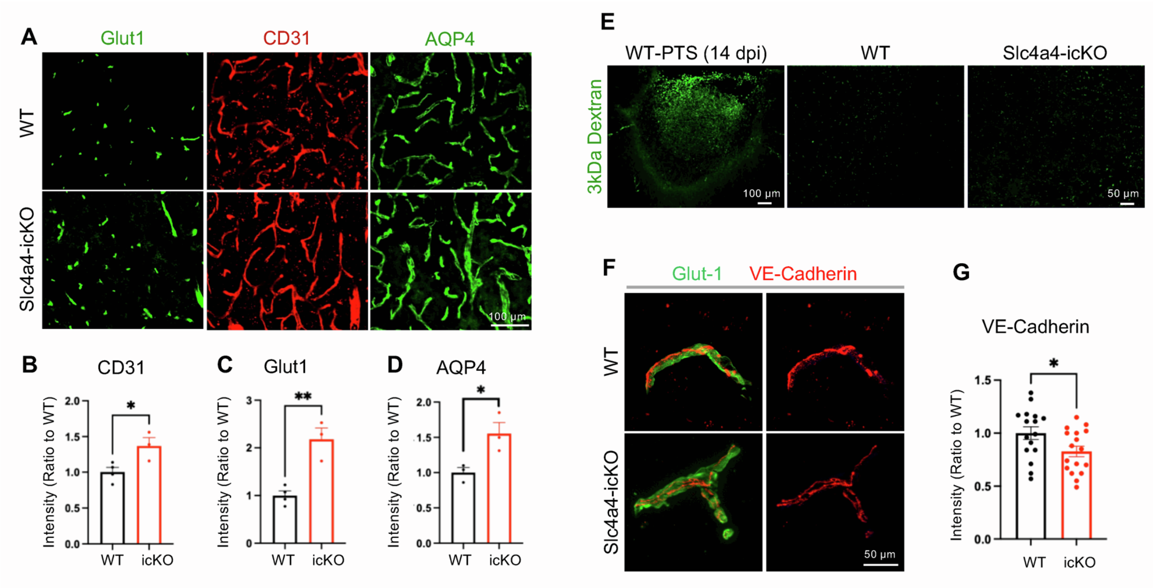

Figure S2. Loss of Slc4a4 increases the expression of endothelial markers but does not induce leakage of 3kDa dextran. (A) Vasculature markers (CD31, Glut1, and AQP4) in Slc4a4-icKO cortices were examined by immunofluorescence staining at P90. **(B-D)** Quantification of endothelial markers (CD31, Glut1, AQP4) based on intensity by immunostaining. Data are presented as mean ± SEM. Each dot represents an individual animal. n = 3-4 animals per group. *p<0.05, **p<0.01 by Student's t-test. **(E)** Representative images of brain sections from n = 3 animals per group after 3kDa FITC-dextran injection. Stroked brains served as a positive control. **(F-G)** Adherent junctional marker VE-Cadherin was examined by immunofluorescence staining at P90. Data are presented as mean ± SEM. Each dot represents an individual image. n =16 vessels collected from 4-5 animals per group. *p<0.05 by Student's t-test.

Figure S3 Related to Figure 3

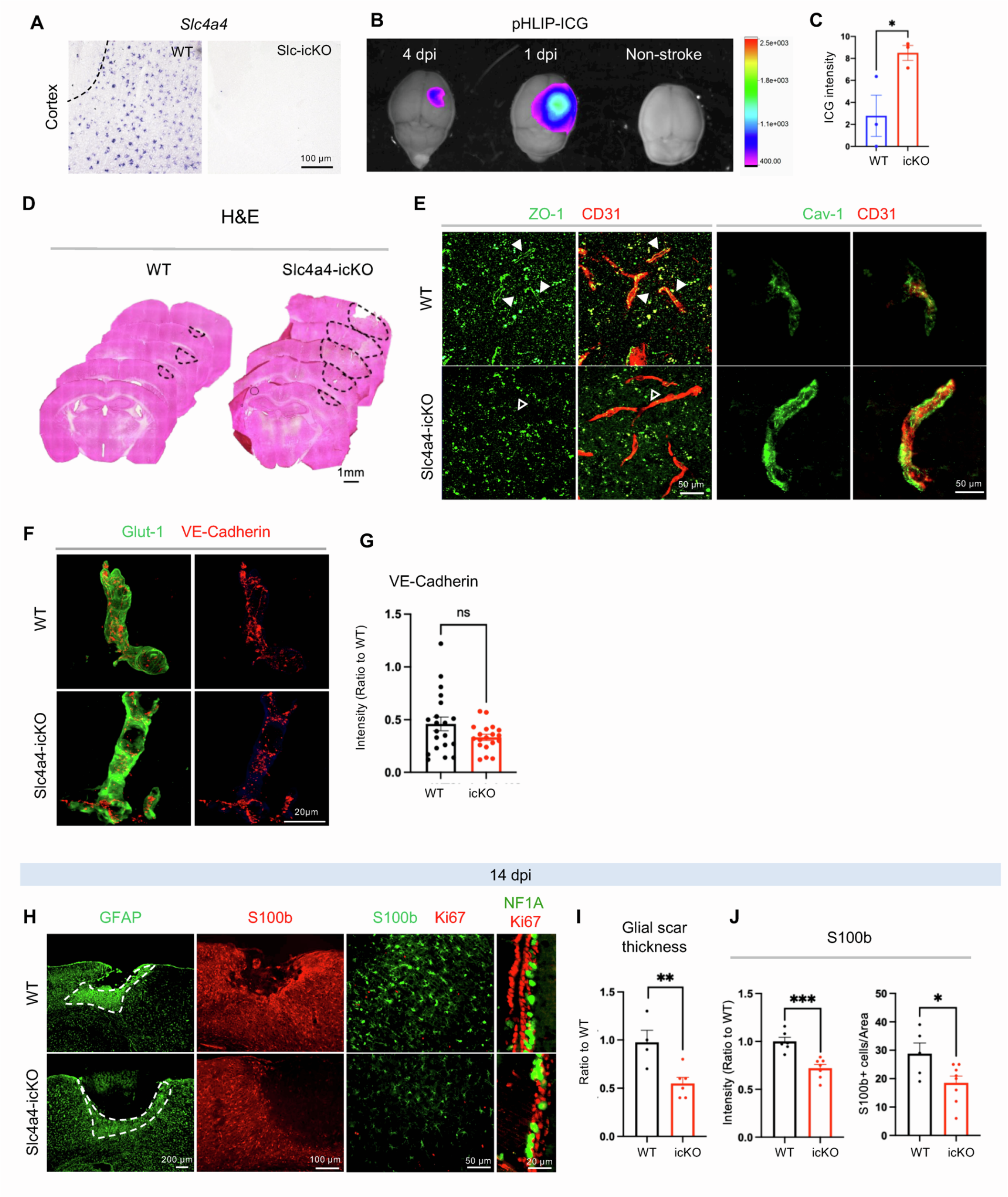

Figure S3. Loss of astrocytic Slc4a4 exacerbates stroke-induced injury and impairs reactive astrogliosis. (A) In situ hybridization confirms the deletion of Slc4a4 in the cortex after stroke at 4 dpi. **(B)** Extracellular pH in the stroke lesions was measured by intraperitoneal injection of pHLIP-ICG dye (1mg/kg). 24 hours after injection (1 and 4 dpi), brains from mice were harvested and imaged using the Bruker Xtreme Imager with 735 nm excitation and 830 nm emission wavelength. **(C)** Quantification of ICG intensity by fluorescence signal detected in WT and Slc4a4-icKO at 1 dpi. Each data point represents an individual animal. n = 3 animals per genotype. *p<0.05 by Student's t-test. **(D)** Representative images of serial brain sections (500µm apart) after H&E staining at 4 dpi.

(E) Representative images of Claudin-5 and Cav-1 at the peri-lesion site of WT and Slc4a4-icKO mice at 4 dpi. Empty arrowheads indicate vessels missing coverage by tight junction proteins. **(F-G)** Adherent junctional marker VE-Cadherin was examined by immunofluorescence staining at 4 dpi. Data are presented as mean ± SEM. Each dot represents an individual image. n = 19-20 vessels collected from 4-5 animals per group. No significance by Student's t-test. **(H)** Immunostaining of reactive astrocyte markers (GFAP, S100b) at the peri-lesion area and SVZ at 14 dpi. S100b+ cells are co-labeled with proliferation marker Ki67 to indicate local astrocyte proliferation. NF1A+ cells are co-labeled with proliferation marker Ki67 to indicate SVZ astrocyte proliferation. **(I)** Quantification of glial thickness based on GFAP staining. Each dot represents an individual animal. n = 4-6 animals per genotype. **p< 0.01 by Student's t-test. **(J)** Quantification of S100b intensity and the number of reactive astrocytes. Data are presented as mean ± SEM. Each dot represents an individual animal. n = 4-6 animals per genotype. *p<0.05, ***p<0.001 by Student's t-test.

Figure S4

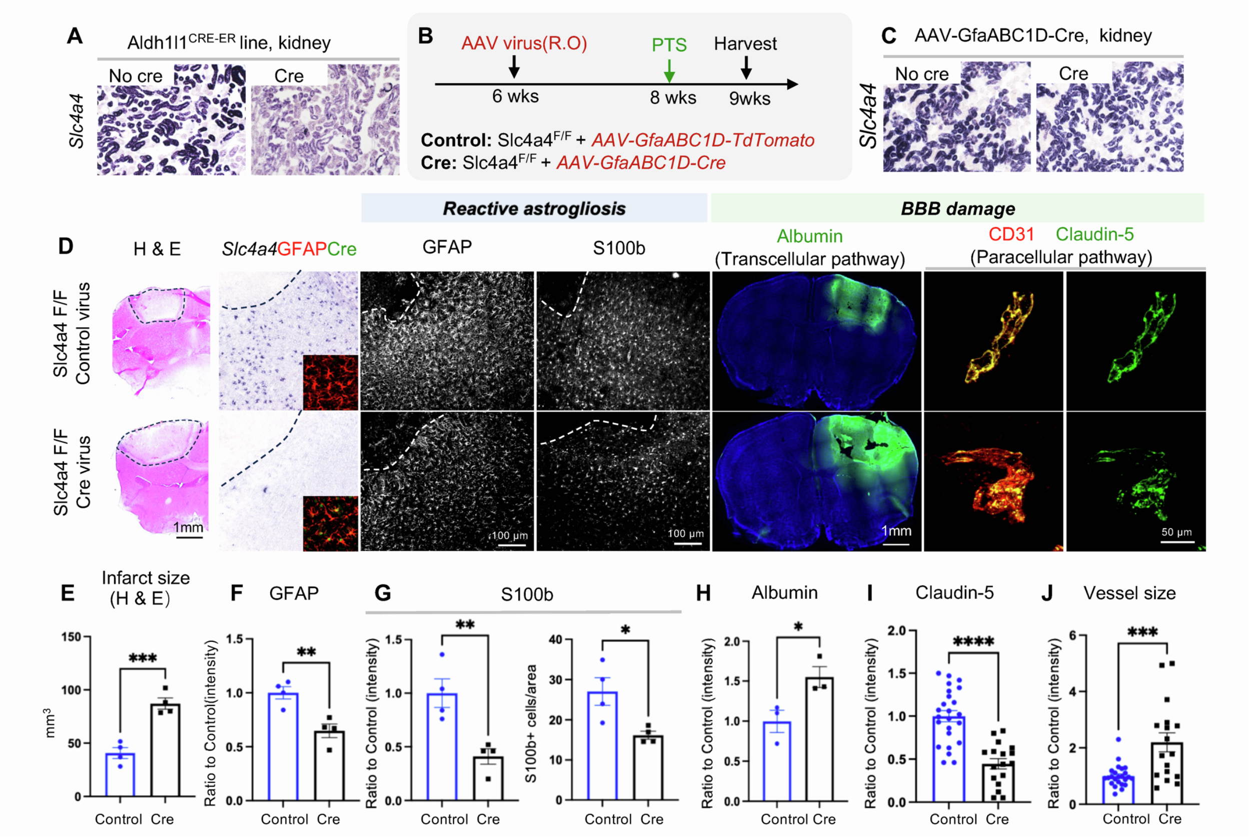

Figure S4. AAV-mediated brain-specific deletion of Slc4a4 dampens reactive gliosis and exacerbates stroke-induced BBB dysfunction. (A) In situ hybridization shows partial deletion of Slc4a4 in the kidney of Slc4a4-icKO mice. **(B-C)** Schematics of AAV-GfaABC1D-Cre virus-mediated Slc4a4 deletion in Slc4a4$^{F/F}$ mice, followed by PTS surgery. AAV-GfaABC1D-TdTomato was used as control. In situ hybridization no changes in kidney Slc4a4 expression in the Cre group. **(D)** Representative images of H&E imaging, in situ hybridization of Slc4a4, immunostaining of Cre, reactive astrocyte marker (GFAP, S100b), albumin leakage, and BBB markers at 4 dpi. **(E)** Quantification of infarct size is based on H&E staining at 4 dpi. Each dot represents an individual animal. n = 4 per group. ***p<0.001 by Student's t-test. **(F)** Quantification of GFAP intensity from immunostaining. Each dot represents an individual animal. n = 4 per group. **p<0.01 by Student's t-test. **(G)** Quantification of S100b intensity and S100b+ cell number from immunostaining. Each dot represents an individual animal. n = 4 per group. *p<0.05, **p<0.01 by Student's t-test. **(H)** Quantification of albumin intensity from immunostaining. Each dot represents an individual animal. n = 3 animals per group. *p<0.05 by Student's t-test. **(I)** Quantification of tight junctional markers Claudin-5 based on their intensity colocalized with CD31 in immunostaining. Each dot represents an individual blood vessel. n = 20-24 blood vessels collected from 4 animals per group. ****p<0.0001 by Student's t-test. **(J)** Quantification of vessel size based on CD31 immunostaining. Each dot represents an individual blood vessel. n = 20-24 blood vessels collected from 4 animals per group ***p<0.001 by Student's t-test. All data are presented as mean ± SEM.

Figure S5 Related to Figure 5

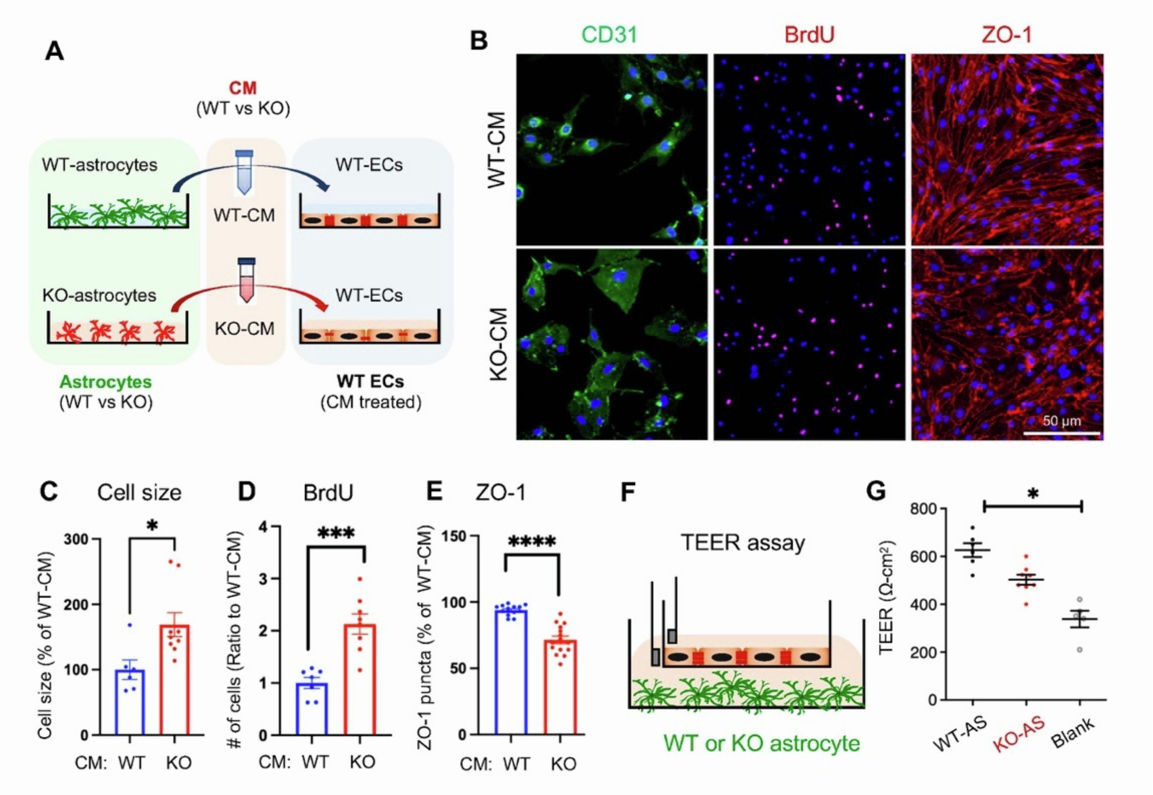

Figure S5. Slc4a4 regulates astrocyte-endothelial interaction via astrocytic secretion factor. (A) Mouse endothelial cell line (bEnd3) was incubated with conditioned media collected from primary WT and Slc4a4 KO astrocytes for 24 hours. **(B-E)** Endothelial cell size was examined by CD31 staining, cell proliferation was examined by BrdU assay, and tight junctional expression was examined by ZO-1 expression in bEnd3 incubated with conditioned media collected from primary WT and Slc4a4 KO astrocytes. Data are presented as mean ± SEM. Each dot represents an individual well. n = 6 wells from three independent cultures. *p<0.05, ***p<0.001, ****p<0.0001 by Student's t-test. **(F)** Experimental setup of the transendothelial electrical resistance (TEER) assay. **(G)** The electro-resistance of the endothelial cell monolayer was measured as an indicator of the permeability of endothelial cells in the TEER assay. Data are presented as mean ± SEM. Wells without cells were used as blank control. Each dot represents an individual well. n = 6 wells from three independent cultures. *p<0.05 by Student's t-test.

Figure S6 Related to Figure 5

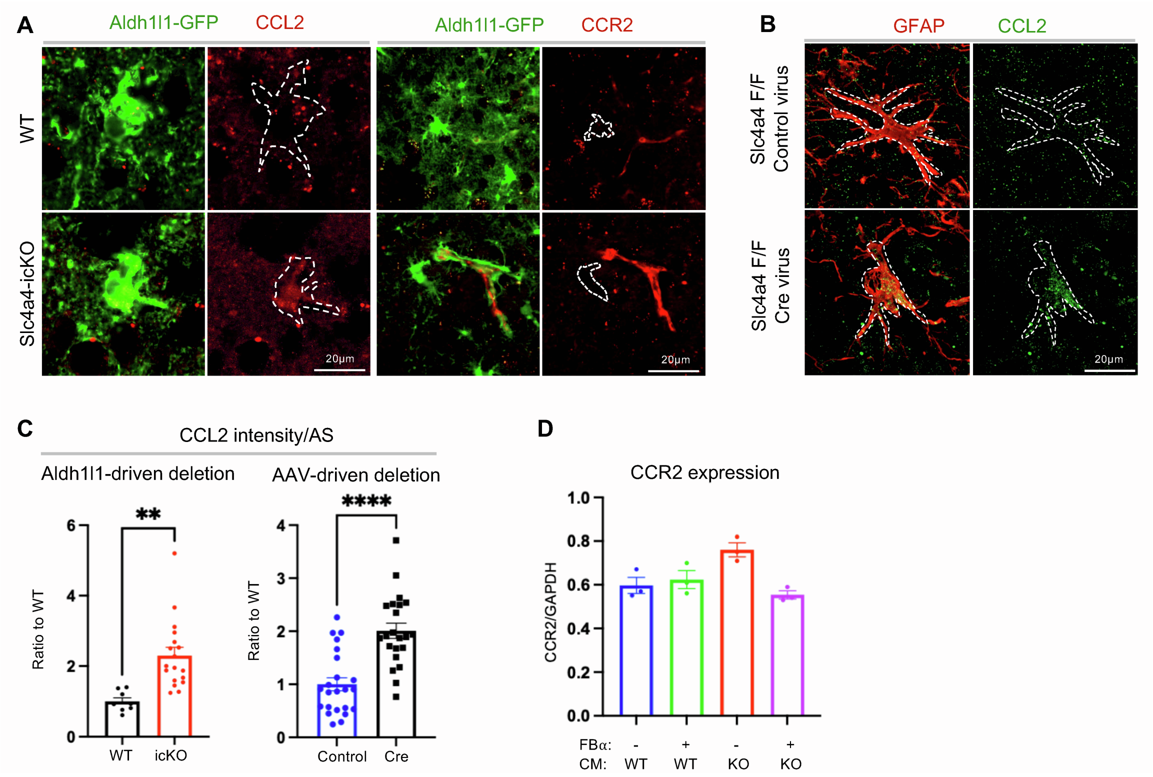

Figure S6. Loss of Slc4a4 upregulates astrocytic CCL2, but not astrocytic CCR2 expression. (A-B) Representative images of astrocytic CCL2 and CCR2 expression from double immunostaining of Aldh1l1-GFP or GFAP and CCL2. **(C)** Quantification of astrocytic CCL2 expression from double immunostaining of Aldh1l1-GFP or GFAP and CCL2. Data are presented as mean ± SEM. Each dot represents an individual astrocyte n = 8-18 cells collected from 3-5 mice per genotype. **$p<0.01$, ****$p<0.0001$ by Student's t-test. **(D)** Western blot quantification of CCR2 expression in bEnd3 cells incubated with WT- and Slc4a4 KO-CM with CCR2 FBα or control IgG. Data are presented as mean ± SEM. Each dot represents each independent culture. n = 3 independent cultures per group. *$p<0.05$, **$p<0.01$ by two-way ANOVA.

Figure S7 Related to Figure 5

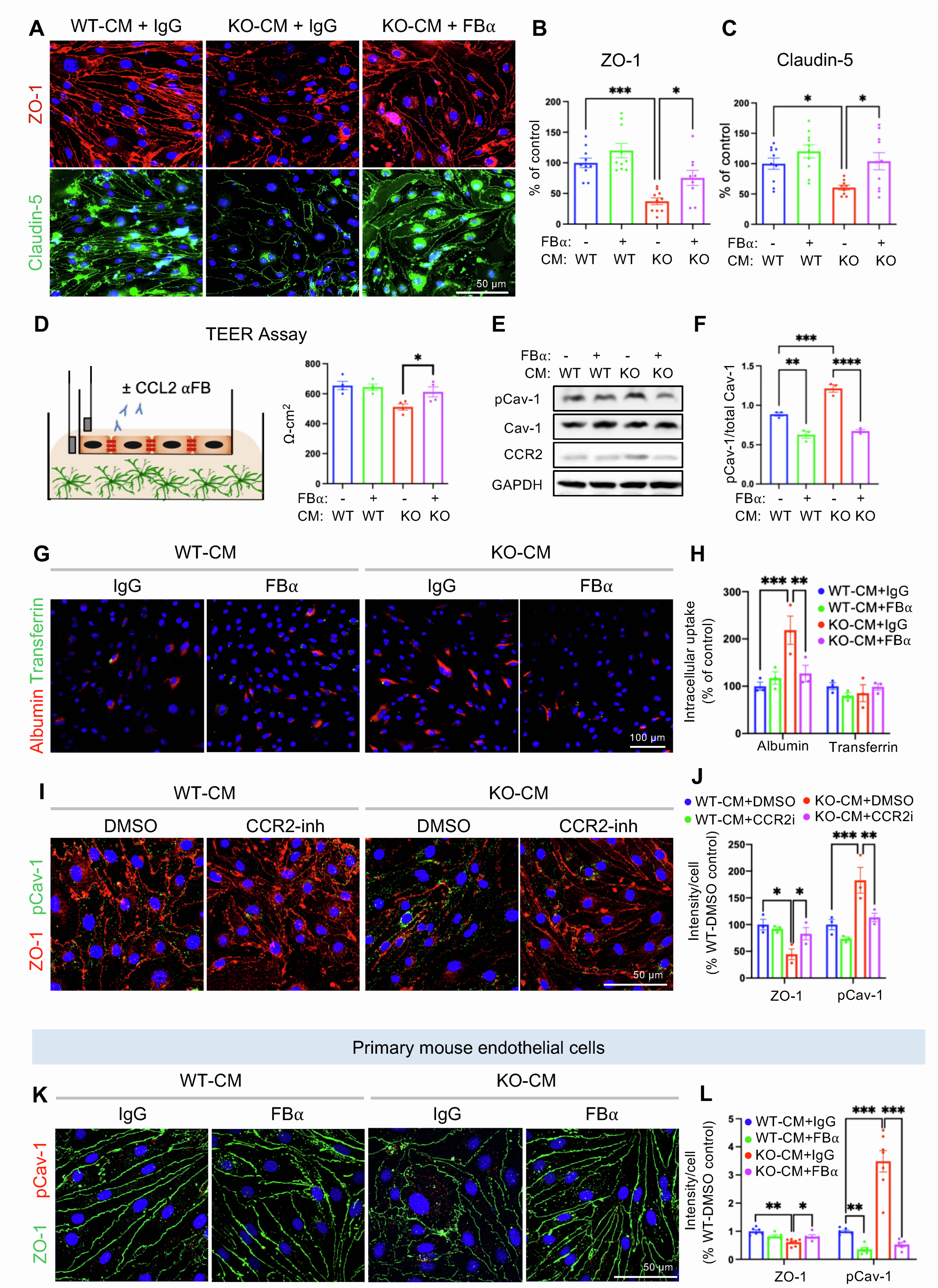

Figure S7. Slc4a4 regulates astrocyte-endothelial interaction via the CCL2-CCR2 axis in vitro. (A-C) Representative immunofluorescence images and quantification of tight junction proteins (ZO-1 and Claudin-5) of bEnd3 cells incubated with WT- or Slc4a4 KO- CM in the presence of control IgG or CCL2-FBα (15 ng/ml) for 24 hours. Each dot represents an individual well. n = 9-10 wells collected from 3 independent experiments per group. *$p<0.05$, ***$p<0.001$ by two-way ANOVA. **(D)** Experimental setup of the transendothelial electrical resistance (TEER) assay. CCL2 blocking antibody was added into the insert with a final concentration of 15 ng/ml. The electro-resistance of the endothelial cell monolayer was measured as an indicator for the permeability of endothelial cells in the TEER assay. Each dot represents each independent culture. n = 4 independent cultures per group. *$p<0.05$, **$p<0.01$ by two-way ANOVA. **(E-F)** Western blot analysis of pCav-1, Cav-1 and CCR2 expression in bEnd3 cells incubated with WT- or Slc4a4 KO-CM with CCR2 FBα or control IgG. Each dot represents an independent culture. n = 3 independent cultures. **$p<0.01$, ***$p<0.001$, ****$p<0.0001$ by two-way ANOVA. **(G-H)** Caveolin- and clathrin-mediated endothelial intracellular uptake was examined by Texas Red conjugated albumin and A488-transferrin, respectively, in bEnd3 cells incubated with WT- or Slc4a4 KO-CM with CCR2 FBα or control IgG. Each dot represents an independent culture. n = 3 independent cultures per group. **$p<0.01$, ***$p<0.001$ by two-way ANOVA. **(I-J)** Paracellular and transcellular endothelial transport in bEnd3 cells incubated with WT- or Slc4a4 KO-CM with CCR2 antagonist RS504393 (10 μM) or control DMSO were examined by immunostaining of ZO-1 and pCav-1. Each dot represents an independent culture. n = 3 cultures per group. *$p<0.05$, **$p<0.01$, ***$p<0.001$ by two-way ANOVA. All data are presented as mean ± SEM. **(K-L)** Paracellular and transcellular endothelial transport in primary mouse endothelial cells incubated with WT- or Slc4a4 KO-CM in the presence of control IgG or CCL2-FBα were examined by immunostaining of ZO-1 and pCav-1. Each dot represents an independent culture. n = 4 cultures per group. *$p<0.05$, **$p<0.01$, ***$p<0.001$ by two-way ANOVA. All data are presented as mean ± SEM.

Figure S8 Related to Figure 6

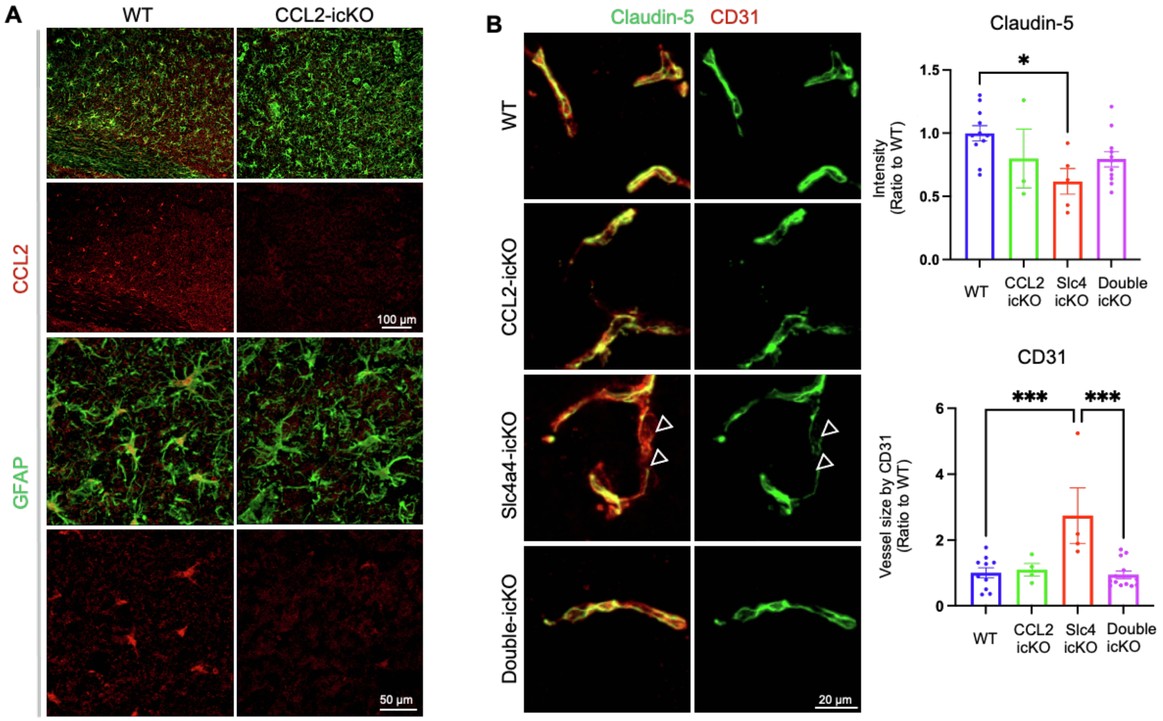

Figure S8 Deletion of astrocytic CCL2 rescues loss of Slc4a4-induced tight junctional marker expression. (A) Confirmation of CCL2 deletion in reactive astrocytes in stroked CCL2-icKO mice by double immunostaining. GFAP was used as a reactive astrocyte marker. **(B)** Representative images and quantification of junctional marker expression (Claudin-5+; CD31+) in the cortex from WT, CCL2-icKO, Slc4a4-icKO, and double-icKO without injury. Data are presented as mean ± SEM. Each dot represents an individual blood vessel. n = 3-11 blood vessels collected from 3-5 animals per group. *$p<0.05$, ***$p<0.001$ by two-way ANOVA.

Figure S9 Related to Figure 6

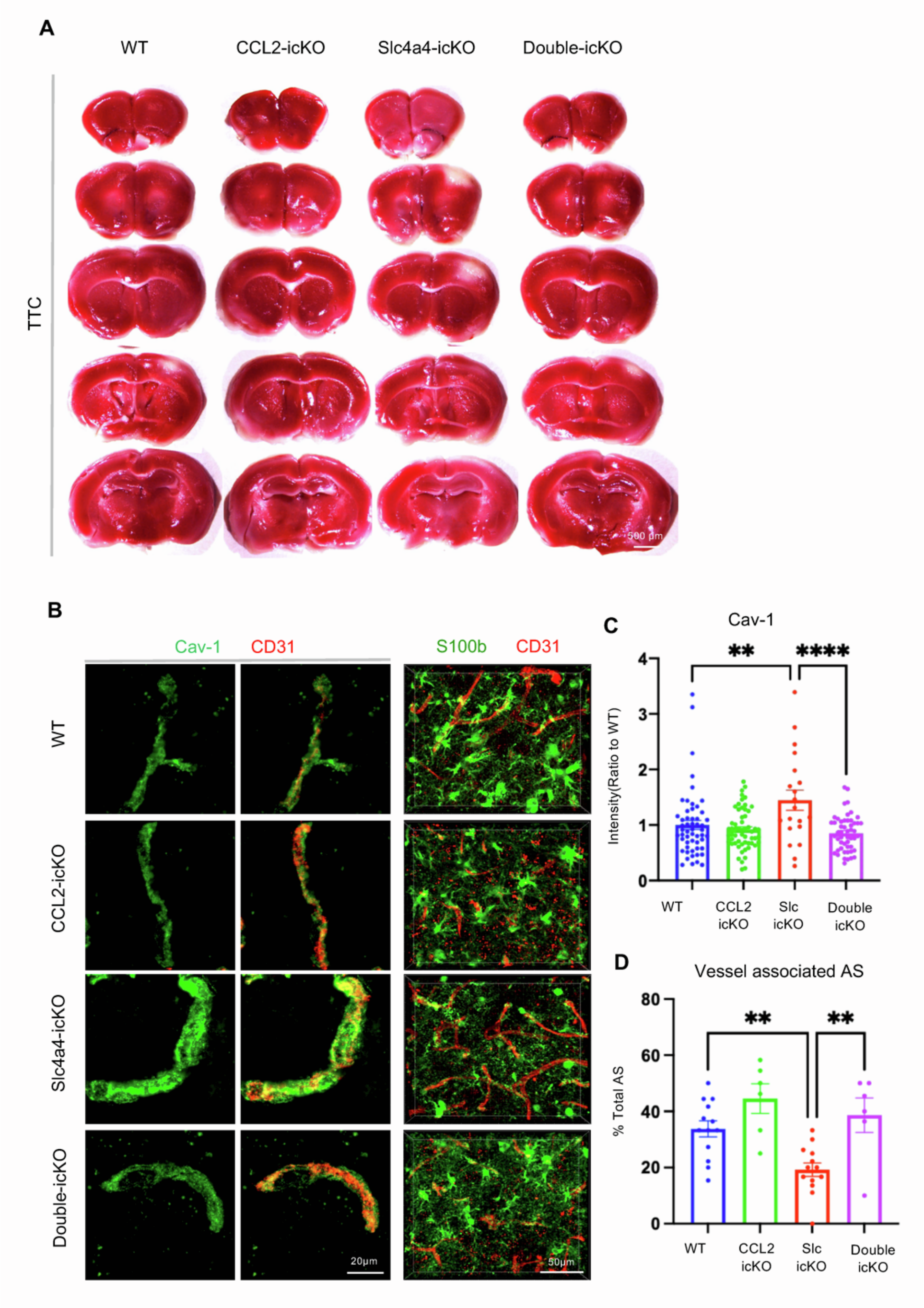

Figure S9. Deletion of astrocyte-derived CCL2 reverses stroke injury exacerbated by loss of Slc4a4. (**A**) Representative images of 2,3,5-Triphenyltetrazolium chloride (TTC) staining at 4 dpi. (**B**) Representative images of endothelial caveolae marker (Cav-1+; CD31+) at peri-lesion area at 4 dpi. Astrocytes are labeled by S100b, and blood vessels are labeled by CD31. **(C)** Quantification of the intensity of Cav-1 colocalized with CD31. Data are presented as mean ± SEM. Each dot represents an individual blood vessel. n = 20-57 blood vessels collected from 4-6 animals per group. **p<0.01,

****p<0.0001 by two-way ANOVA. **(D)** Quantification of vessel-associated astrocytes by S100b and CD31 double immunostaining. Vessel-associated astrocytes are defined as those astrocytes whose somas occupy vascular territory. Data are presented as mean ± SEM. Each dot represents an individual animal. n = 6-13 sections collected from 4-6 animals per group. **p<0.01 by two-way ANOVA.

Figure S10

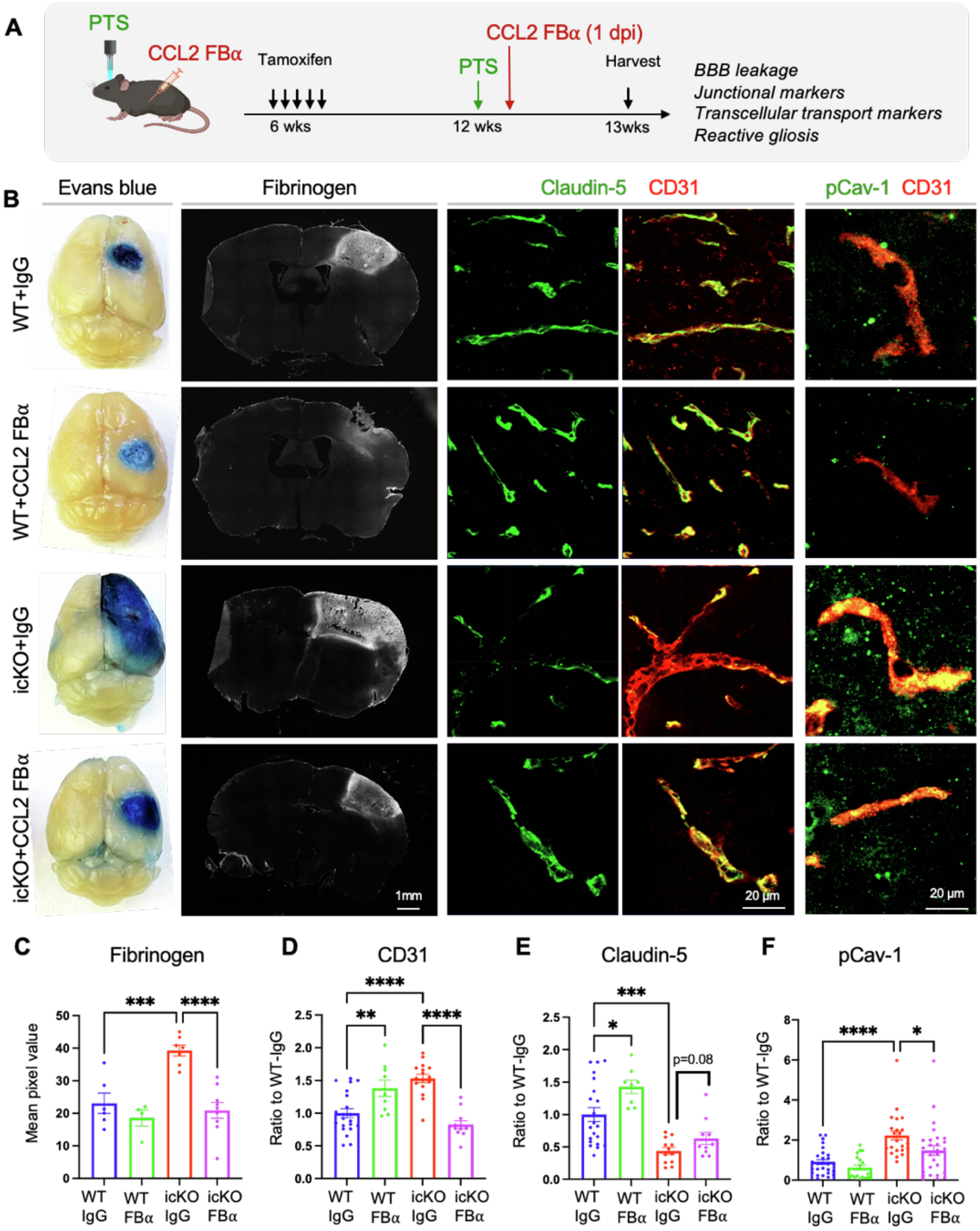

Figure S10. Pharmacological inhibition of CCL2 rescues loss of Slc4a4-induced exacerbated BBB damage after ischemic stroke. (A) Experimental scheme of the PTS induction in WT and Slc4a4-icKO mice, followed by intraperitoneal injection of CCL2 functional blocking antibody (CCL2-FBα, 0.5 mg/kg) at 1 dpi. Brains were then harvested and analyzed at 4 dpi. **(B)** Representative images of protein leakage (Evans blue,

fibrinogen), endothelial junctional marker expression (Claudin-5+; CD31+) and endothelial pCav-1 expression (pCav-1+; CD31+) at the peri-lesion area. **(C)** Quantification of fibrinogen intensity from immunostaining. Each dot represents an individual animal. n = 4-9 animals per group. ***p<0.001, ****p<0.0001 by two-way ANOVA. **(D)** Quantification of CD31 intensity from immunostaining. Each dot represents an individual blood vessel. n = 8-21 blood vessels collected from 4-5 animals per group. **p<0.01 ***p<0.001, ****p<0.0001 by two-way ANOVA. **(E)** Quantification of the intensity of Claudin-5 colocalized with CD31. Data are presented as mean ± SEM. Each dot represents an individual blood vessel. n = 8- 21 blood vessels collected from 4-5 animals per group. *p<0.05, ***p<0.001 by two-way ANOVA. **(F)** Quantification of the intensity of pCav-1 colocalized with CD31. n = 17-27 blood vessels collected from 4-5 animals per group. *p<0.05, ***p<0.001 by two-way ANOVA. All data are presented as mean ± SEM.

Figure S11 Related to Figure 7

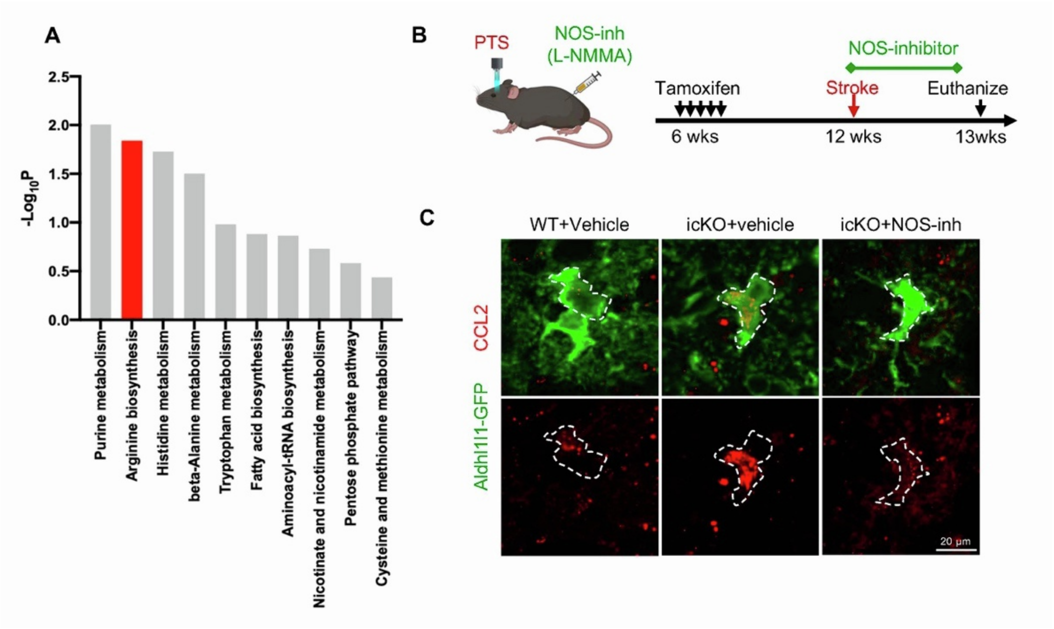

Figure S11. Inhibition of pan-NOS rescues exacerbated BBB leakage and increased astrocytic CCL2 in Slc4a4-icKO after stroke (A) Cortices were collected from WT and Slc4a4-icKO mice and subjected to unbiased metabolomic analysis, followed by pathway analysis. **(B)** Experimental scheme of the PTS induction in WT and Slc4a4-icKO mice, followed by daily intraperitoneal injection of a pan-NOS inhibitor (L-NMMA) from 1-3 dpi. Brains were then harvested and analyzed at 4 dpi. **(C)** Representative images of CCL2 colocalized with Aldh1l1-GFP at the peri-lesion area.

Figure S12 Related to Figure 7

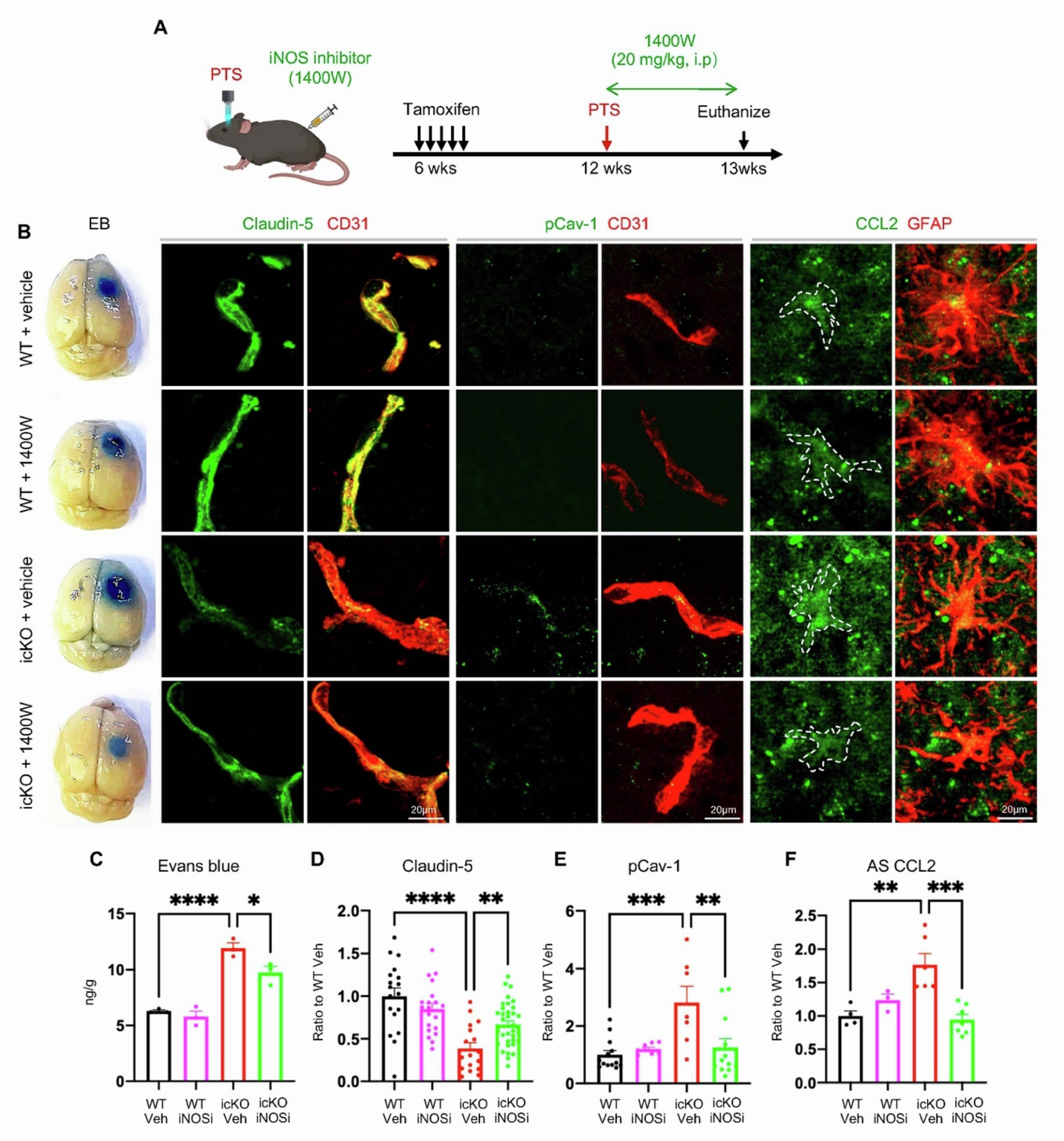

Figure S12. Inhibition of iNOS partially rescues exacerbated BBB leakage and increased astrocytic CCL2 in Slc4a4-icKO after stroke (A) Experimental scheme of the PTS induction in WT and Slc4a4-icKO mice, followed by daily intraperitoneal injection of an iNOS inhibitor (1400W) from 1-3 dpi. Brains were then harvested and analyzed at 4 dpi. **(B)** Representative images of Evans blue leakage, Claudin-5, pCav-1 colocalized with CD31, and CCL2 colocalized with Aldh1l1-GFP at the peri-lesion area. **(C)** Quantification of Evans blue leakage by colorimetric assay from stroked brains at 4 dpi.

Data are presented as mean ± SEM. Each dot represents an individual animal. n = 3 animals per group. *p<0.05, ****p<0.0001 by two-way ANOVA. **(D-E)** Quantification of the intensity of Claudin-5 or Cav-1 colocalized with CD31. Data are presented as mean ± SEM. Each dot represents an individual blood vessel. n = 8-36 blood vessels collected from 3-5 animals per group. **p<0.01, ***p<0.001, ****p<0.0001 by two-way ANOVA. **(F)** Quantification of the intensity CCL2 colocalized with GFAP. Data are presented as mean ± SEM. Each dot represents an individual animal. n = 3-7 animals per group. **p<0.01, ***p<0.001, ****p<0.0001 by two-way ANOVA
